# Spikes as perturbations of resonant neural circuits: an RLC framework with testable predictions

**DOI:** 10.3389/fncom.2026.1846697

**Published:** 2026-07-08

**Authors:** Jeremy Sender, Yi-Ping Phoebe Chen

**Affiliations:** Department of Computer Science and Information Technology, La Trobe University, Melbourne, VIC, Australia

**Keywords:** I_h_, membrane inductance, neural computation, neuromorphic computing, neuronal impedance, phase-resonance, resonate-and-fire, RLC circuit model

## Abstract

Computational neuron models commonly reduce subthreshold membrane dynamics to a leaky RC integrator, confining computation to the spike and treating the inter-spike trajectory as passive decay. Yet impedance measurements and channel-specific analyses show that many excitable membranes have band-pass, inductance-like impedance profiles with a tuneable resonant peak — dynamics a first-order RC model cannot represent. This paper develops a spike-as-perturbation framework in which spikes act as impulse perturbations that launch regime-dependent transient trajectories in an equivalent parallel RLC membrane. We define the biological grounding and domain of validity of the reduction, show that post-perturbation RLC ringdowns carry circuit-identity and perturbation-timing state variables not available to a single matched first-order RC element without added delays, recurrence, or extra state variables, and demonstrate a concrete primitive — phase-based temporal discrimination — together with a spike-timing readout that makes it network-visible. A closed-form comparison (*Q* = 2, τRC = 25 ms, f_0_ = 10 Hz) gives an RLC sensitivity half-life of 44 ms versus 17 ms for a matched RC decay. We identify the quality factor Q — strongly shaped by I_h_ and related slow conductances — as a candidate neuromodulatory control variable that fixes the membrane pole radius and hence its transient (short-term) memory horizon, the biological analogue of selectivity in machine state-space models. We do not claim a universal *in vivo* phase code: a simulation under high-conductance bombardment shows that the readout is reliable in quiescent, high input-resistance states and is suppressed as conductance loading drives the effective Q below the underdamped threshold. We derive four falsifiable predictions spanning cellular, network, decoding, and population levels, and present the argument in three tiers of decreasing evidential support.

## 1 Introduction

Computational neuron models commonly reduce subthreshold dynamics to a leaky RC integrator, treating the spike as the primary computational event and the membrane as a passive substrate between spikes. This framing has been enormously productive, but it encodes a specific assumption: that the inter-spike membrane trajectory is a monotonic exponential decay carrying no structured information beyond the scalar time constant τ = RC. Is this assumption always justified?

This paper argues otherwise. It proposes that excitable neural membranes, in appropriate regimes, function as resonant dynamical elements whose post-perturbation trajectories carry structured state information that may itself participate in computation. The core thesis is:

*Spike-triggered synaptic events can be approximated as impulse-like perturbations of the linear subthreshold dynamics when their timescale is short relative to the resonant period, launching structured state trajectories in resonant neural tissue. Those post-perturbation trajectories may carry decodable state information through their phase, damping, and frequency structure, providing an additional subthreshold dynamical variable for temporal discrimination and state-dependent spike timing. A cognitive interpretation of these dynamics is deferred to section “8.5 Speculative extensions and testable predictions” and treated there as a clearly labeled speculative extension.*

Unlike prior resonate-and-fire models (Izhikevich, 2001) that focus on how resonance shapes spike generation — the pre-spike subthreshold dynamics that determine threshold crossing — this framework extends the computational role of resonance to post-spike dynamics: the structured ringdown trajectory launched by a spike in a postsynaptic resonant membrane. Izhikevich (2001) demonstrated that complex-eigenvalue subthreshold dynamics produce frequency preference, post-inhibitory rebound, and resonant amplification of periodic inputs. The present contribution asks a distinct question: given that resonant postsynaptic membranes exist, what subthreshold dynamical processing does the post-perturbation ringdown itself perform between spikes? The answer — that phase, damping, and frequency structure of the ringdown carry circuit-identity and timing information absent from RC decays — is the specific claim not addressed by resonate-and-fire models. A simulation analysis (section “4.5 Simulation: temporal sensitivity of RC vs. RLC ringdowns”) quantifies this advantage at representative biological parameters (*Q* = 2, τRC = 25 ms, f_0_ = 10 Hz) using an analytic sensitivity envelope metric: under these assumptions, temporal sensitivity in RLC ringdowns persists 2.5× longer than in matched first-order RC decays, with model-derived sensitivity ratios exceeding 17× at 100 ms readout latency (*Q* = 2 specific).

The biophysical motivation is direct. Classic longitudinal impedance measurements show that most axons exhibit peak impedance at low frequency, with reactance vanishing between 150 and 300 Hz, establishing a reactive component with non-trivial frequency dependence (Cole and Baker, 1941). Phenomenological analysis identifies inductance-like elements whose effective parameters vary with membrane potential and channel state (Mauro et al., 1970). Modern channel-specific impedance measurements in mammalian neurons confirm that specific conductances — notably I_h_ — contribute a location-dependent and adaptive inductive component with theta-range phase-leading behavior (Narayanan and Johnston, 2008).

This paper connects four threads that have remained largely separate in the literature: (i) direct impedance evidence for inductive behavior (Cole and Baker, 1941; Cole and Curtis, 1939; Sabah, 2000); (ii) the phenomenological, regime-dependent nature of effective inductance (Mauro et al., 1970); (iii) the distinction between amplitude resonance and phase-resonance as separable response properties (Rotstein, 2014); and (iv) the non-linear transduction requirement that converts resonant subthreshold dynamics into frequency-selective information transfer (Blankenburg et al., 2015). By unifying these threads within a single equivalent-circuit framework, the paper identifies a concrete computational primitive — phase-based temporal discrimination — that is not straightforwardly available in matched first-order RC models, and derives four testable predictions.

The argument is presented in three tiers of decreasing evidential support. Tier 1 (well-supported substrate): neural membranes exhibit resonant dynamics in appropriate regimes, compact RLC equivalent circuits capture the essential impedance structure, and specific ionic mechanisms generate the inductive component (Cole and Baker, 1941; Fenollosa and Bisquert, 2025; Hutcheon et al., 1996; Mauro et al., 1970; Narayanan and Johnston, 2008; Rotstein, 2014; Ulrich, 2014). Tier 2 (plausible inference): the structured post-perturbation trajectory of an RLC membrane carries richer state information than an RC decay, and this enrichment may provide computational primitives beyond amplitude coding. Tier 3 (speculative extension): these dynamics may underpin aspects of representation, memory, and attention at the cognitive level.

The paper is organized as follows. Section “2 Biophysical foundations” develops impedance-to-circuit foundations, introduces a parallel RLC equivalent circuit, and defines the biological grounding and domain of validity of the reduction. Section “3 Evidence for inductance and resonance” reviews evidence for inductive membrane components and resonance phenomena. Section “4 The spike as perturbation” presents the spike-as-perturbation interpretation, including a representational comparison, a worked computational primitive, a simulation analysis, and a sensitivity analysis. Section “5 Readout — subthreshold trajectories become spike-timing modulation” closes the readout gap by showing how subthreshold trajectories become spike-timing modulation. Section “6 Relationship to existing neuron models” positions the framework relative to existing neuron models. Section “7 Population dynamics: non-linear transduction” establishes the population-level non-linear transduction requirement. Section “8 Discussion” discusses limitations, testable predictions, neuromorphic implications, and speculative extensions.

Contribution: This paper does not claim that RLC membrane models, subthreshold resonance, or resonate-and-fire dynamics are new. Its contributions are three: the interpretation of perturbation-induced resonant transients as a readable state variable; an explicit spike-timing readout mechanism that makes that variable network-visible; and the identification of the quality factor Q as a biophysical, neuromodulatory control parameter for the membrane’s transient (short-term) memory horizon — its state-persistence time, not memory in the synaptic or cognitive sense — providing the runtime pole-radius control that machine state-space models obtain only through training. The empirical content is the equivalent-circuit substrate, the phase-discrimination primitive with its readability condition, and four falsifiable predictions; the cognitive reading (section “8.5 Speculative extensions and testable predictions) is a clearly labeled speculative extension.

## 2 Biophysical foundations

### 2.1 Classic impedance measurements

Classic axonal impedance measurements provide direct empirical motivation for incorporating inductive reactance into membrane descriptions (Cole and Baker, 1941). Longitudinal measurements show that for most axons, impedance peaked at low frequency and reactance vanished between 150 and 300 Hz, indicating the presence of a reactive component beyond simple capacitance (Cole and Curtis, 1939; Sabah, 2000). These measurements are not easily dismissed as artifacts: the frequency-dependent structure is consistent across preparations and is reproduced by models incorporating an inductive element in parallel with the membrane capacitance and resistance.

These data also provide a quantitative anchor for the equivalent circuit. For a square centimeter of membrane, Cole and Baker reported a capacitance of 1 μF with dielectric loss, shunted by a 400 Ω resistance in series with a 0.2 H inductance — an explicit inductive element required to account for the frequency response (Cole and Baker, 1941). Later work (Sabah, 2000) confirmed that the impedance locus requires more than a simple RC description and that a membrane-localized reactive component is present.

### 2.2 Phenomenological inductance and regime dependence

A key refinement is that inductance-like behavior in excitable membranes can be phenomenological and state-dependent rather than a fixed passive component (Mauro et al., 1970). Mauro et al. (1970) described the behavior as arising from time-variant conductances whose kinetics mimic inductive reactance in the impedance domain, establishing an equivalent-circuit approach that treats such behavior as a valid and useful engineering description without requiring a literal inductor.

Critically, this account includes voltage dependence. At relatively hyperpolarized levels, the impedance degenerates to an RC system — inductance-like signatures can disappear depending on operating point and channel state (Mauro et al., 1970). Complementary work supports the principle that subthreshold resonance and inductive signatures in biological neurons are conditional phenomena, arising from the interplay of specific voltage-gated conductances rather than from a single passive element (Heys et al., 2010; Narayanan and Johnston, 2008). This regime dependence is not a weakness of the framework but a feature: it predicts that the computational properties described in this paper should be expressed only in specific biophysical states, providing a natural mechanism for state-dependent computation.

### 2.3 The parallel RLC equivalent circuit

**FIGURE 1:**
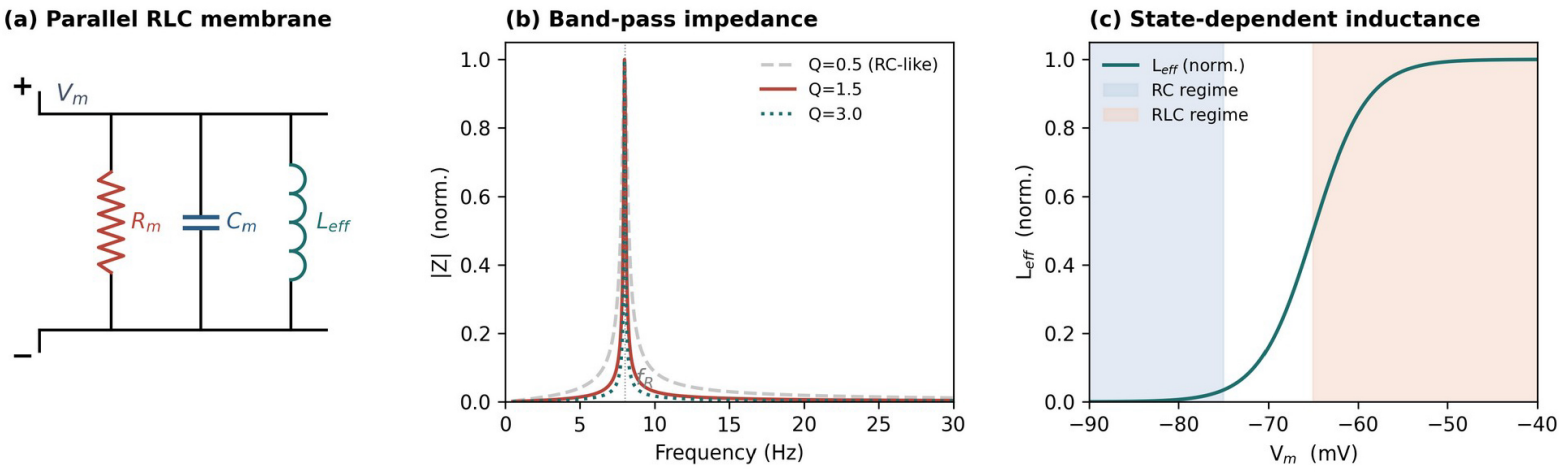
Parallel RLC membrane element and its frequency- and state-dependent behaviour. **(a)** Parallel RLC equivalent circuit of the membrane, with membrane resistance R_m_, capacitance C_m_, and effective inductance L_eff_. **(b)** Band-pass impedance profile for three quality factors (*Q* = 0.5 broadest, *Q* = 1.5, *Q* = 3.0 narrowest). **(c)** State-dependent effective inductance versus membrane voltage V_m_, showing the transition between the RC regime (hyperpolarised) and the RLC regime (depolarised). Effective impedance degenerates to a first-order RC system at hyperpolarised potentials [see Mauro et al. (1970) for the measured hyperpolarised trace]. Biological subthreshold resonance occurs in the 1.20 Hz range (Hutcheon et al., 1996; Rotstein, 2014; Ulrich, 2014) with Q ≈ 1.3 (Vera et al., 2020). Schematic symbols use the standard zigzag resistor, parallel-plate capacitor, and coiled inductor.

The modeling stance adopted here is that excitable membranes can be treated as equivalent circuits whose frequency-dependent impedance is represented using elementary components (Figure 1)— while explicitly distinguishing equivalence from microscopic identity (Cole and Baker, 1941; Fenollosa and Bisquert, 2025; Mauro et al., 1970). The impedance of a parallel RLC element is Equation 1:


Z⁢(s)=1/(1/R+s⁢C+1/(s⁢L)) (1)

This is used as an equivalent-circuit scaffold rather than a claim that a unique physical inductor exists in all neural tissue (Fenollosa and Bisquert, 2025; Mauro et al., 1970). The natural frequency of an ideal LC sub-circuit is Equation 2:


$$\omega_{0}=1/\sqrt{\left(L\,C\right)}$$ (2)

The quality factor for parallel resonance is Equation 3:


$$Q=R\,\sqrt{\left(C/L\right)}$$ (3)

For an underdamped parallel RLC element driven by an impulse from rest, the ringdown is:


$$v\,\left(t\right)=\left(q_{0}/C\right)\cdot e^{\wedge }\,\left(-\alpha \,t\right)\cdot \left[c\,o\,s\,\left(\omega \,\_\,d\,t\right)-\left(\alpha /\omega \,\_\,d\right)\cdot s\,i\,n\,\left(\omega \,\_\,d\,t\right)\right]$$ (4)

Here α = 1/(2RC) is the decay rate and ω_d = √(ω_*0*_^2^ −α^2^) is the damped oscillation frequency. Both α and ω_d are circuit-identity parameters, determined by R, L, and C; they characterize the neuron’s resonant regime rather than the perturbation that elicited the response. Equation 4 treats the spike as a current impulse i(t) = *V*_*0*_⋅δ(t − t_k) applied at spike times t_k that deposits charge q0, instantaneously displacing the membrane voltage by q0/C while the inductor current I_L remains continuous — a fixed displacement of the state vector (V, I_L). Here q0/C has units of volts and the bracketed term is dimensionless, so the expression is dimensionally consistent. Because that term combines a cosine and a sine at the same frequency, Equation 4 is equivalently a single damped sinusoid with an intrinsic phase, which motivates the phase-shifted form of Equation 5. In the lightly damped regime α≪ω_d (Q ≫ 1/2) the sine correction is small and the response is dominated by (q0/C) e∧(−αt) cos(ω_d t).

When a perturbation arrives during an ongoing oscillatory state rather than from rest, the resulting trajectory takes the more general form:


$$v\,\left(t\right)=A\cdot e^{\wedge }\,\left(-\alpha \,t\right)\cdot s\,i\,n\,\left(\omega \,\_\,d\,t+\varphi \right)$$ (5)

where A absorbs both the perturbation amplitude and the pre-existing oscillatory state at the moment of arrival, and the phase offset φ encodes the timing of the perturbation relative to the ongoing oscillation. This phase term is the perturbation-timing parameter: it carries information about when the perturbation occurred relative to the resonant cycle — information that has no analogue in the RC response, where phase is undefined.

Biological resonant neurons operate in a heavily damped regime (Q ≈ 1–3, as reported by Rotstein (2014), Vera et al. (2020); see (Narayanan and Johnston, 2008) for the conductance basis of this range). At *Q* = 1, the ringdown crosses zero once before decaying to noise — limited but non-zero additional information capacity. At *Q* = 3, several oscillatory cycles are available before the transient decays, offering correspondingly richer temporal structure for readout by downstream circuits.

### 2.4 Biological grounding and domain of validity

The parallel RLC circuit is not merely a metaphor; in the small-signal regime, its elements map onto measurable effective biophysical quantities. The capacitance C reflects the specific membrane capacitance of about 1 μF/cm^2^, a value preserved across taxa for over a billion years and treated as the most invariant electrical parameter of the cell membrane (Gentet et al., 2000). The conductance 1/R reflects the linearized leak around the resting attractor. The inductance L is the kinetic mass of slow voltage-gated currents. I_h_ and IM are the canonical sources. Their finite activation time means that a voltage change produces a delayed opposing current — the textbook signature of an inductor (Mauro et al., 1970; Narayanan and Johnston, 2008). Recent work extends this picture to myelin (Wang et al., 2021), where helical wrapping and piezoelectric contributions add a structural inductance term.

The reduction from a full Hodgkin–Huxley membrane to a parallel RLC is a small-signal linearization around a stable resting point. We are not modeling the action potential itself. The AP is the non-linear excursion that lifts the trajectory out of the linear basin. We are modeling the subthreshold trajectory between APs, where conductances are weakly perturbed and a Taylor expansion of their gating dynamics holds. This is the same approximation under which Mauro derived the original RLC equivalent (Mauro et al., 1970). Hutcheon and Yarom (2000) showed it captures the impedance profile of real cortical and thalamic neurons across the 1–20 Hz band.

The framework has explicit boundaries. It applies when the membrane sits in a stable equilibrium with at least one slow current carrying a non-trivial inductive component. Recent network-level analyses show that history-dependent computation in inhibition-stabilized attractor networks requires Q > 0.5 (Hilty and Miller, 2026); below this threshold the dynamics collapse to overdamped RC behavior and the framework reduces to the standard leaky integrator. Above the threshold the second-order dynamics emerge and the framework applies. The transition is continuous, not categorical, and we treat the RC neuron as the Q → 0 limit of the present model rather than as a separate object.

Three further constraints bound the regime of validity. First, the amplitude must remain small enough that gating non-linearities are negligible. Inputs of a few millivolts satisfy this; persistent depolarizations near spike threshold do not. Second, the resonant timescale must be slow compared with the AP itself. For I_h_ and IM kinetics this is satisfied: τ≈ 10–100 ms versus AP widths near 1 ms. Third, the framework treats spikes as instantaneous perturbations of the linear subthreshold dynamics. We make this assumption explicit in Equation 4 and discuss its relationship to the reset rules of resonate-and-fire models in section “6 Relationship to existing neuron models”.

Parameter choices throughout the paper are taken from published recordings. Membrane time constants of 10–30 ms come from Koch (1999). Resonance peaks at 2–10 Hz are reported by Hutcheon et al. (1996) and confirmed across multiple preparations (Ulrich, 2014). The well-supported biological quality factor lies in the range Q ≈ 1–3 (Rotstein, 2014; Vera et al., 2020); higher values up to Q ≈ 8 are reported in particular cell types and recording conditions (Vera et al., 2020) but lie outside the core biological claim. Where this paper uses Q > 3 — in the latency, two-spike, and population analyses of section “4.6 Sensitivity analysis: latency, synaptic jitter, and frequency tuning,” “5 Readout — subthreshold trajectories become spike-timing modulation,” “7 Population dynamics: non-linear transduction” — these are labeled explicitly as exploratory high-Q conditions chosen to make the second-order behavior visible, not as the central biological regime, and the sensitivity to this choice is reported in section “4.6 Sensitivity analysis: latency, synaptic jitter, and frequency tuning.”

## 3 Evidence for inductance and resonance

### 3.1 Active conductances and inductance-like behavior

**FIGURE 2:**
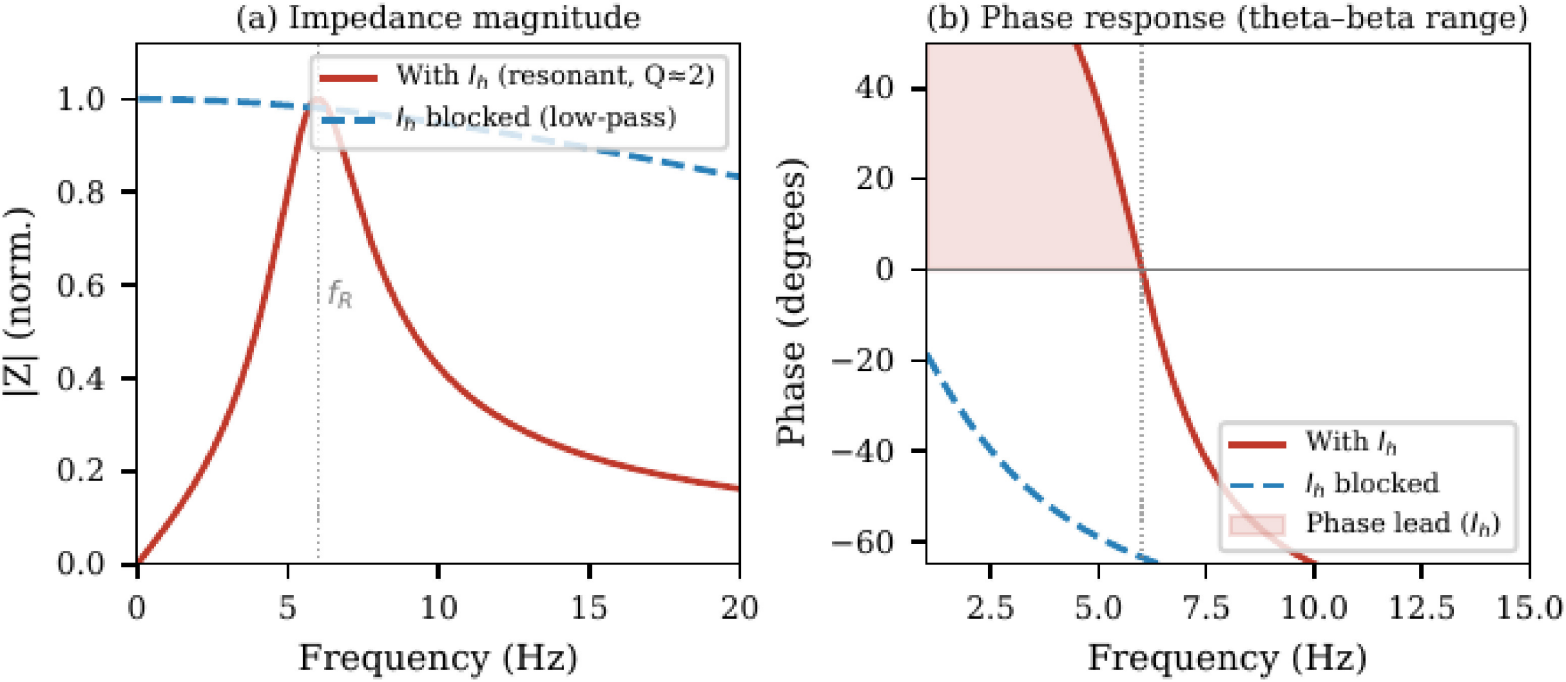
Conductance-specific origin of inductance-like behaviour. **(a)** Impedance magnitude versus frequency: the resonant response with I_h_ (Q ≈ 2) shows a band-pass peak, whereas with I_h_ blocked the response is low-pass. **(b)** Phase response over the theta to beta range: the I_h_ condition produces a phase lead at low frequencies (shaded) before a phase lag, whereas the I_h_-blocked condition decreases monotonically. H channels contribute a location-dependent inductive component and introduce theta-range apparent negative delay in voltage response relative to injected current (Narayanan and Johnston, 2008).

Modern impedance analyses bridge phenomenological inductance to specific ion channels in mammalian neurons (Narayanan and Johnston, 2008). In CA1 pyramidal neurons, h channels contribute a location-dependent and adaptive inductive component to input impedance (Figure 2), supporting the interpretation that the inductive element in the equivalent circuit has a concrete biophysical substrate — one whose parameters can be modulated by experience and neuromodulatory state.

These results complement the classic claim that inductive reactance is a property of the axon, while emphasizing that multiple mechanisms can generate inductive signatures across preparations (Cole and Baker, 1941; Narayanan and Johnston, 2008). Together, these results support interpreting the effective inductance in the equivalent circuit as grounded in identifiable, measurable, and manipulable ionic conductances — while not a single passive component, it has a concrete biophysical substrate.

### 3.2 Subthreshold resonance

Subthreshold resonance is the ability of neurons to exhibit a peak voltage amplitude response to oscillatory input at a preferred non-zero frequency (Rotstein, 2014). In juvenile rat sensorimotor cortical neurons, whole-cell recordings show low-frequency resonance with sensitivity to pharmacological manipulation of I_h_ (Hutcheon et al., 1996). Critically, subthreshold resonance is the steady-state amplitude response to sinusoidal input — not autonomous post-perturbation ringing. The two phenomena share the same underlying RLC structure but reflect different driving conditions: resonance measures the system’s response to continuous oscillatory drive, while the post-perturbation ringing of section “4 The spike as perturbation” is its impulse response. Resonance interacts with active conductances to produce frequency-selective amplification that persists under a range of physiological conditions.

Thalamic evidence directly links impedance resonance to preferred-frequency spike generation under weak suprathreshold drive (Ulrich, 2014). Thalamic reticular nucleus cells show a characteristic resonance at 1.7 Hz at resting membrane potential. Small suprathreshold inputs at 1.7 Hz evoke reliable spiking; inputs at other frequencies do not. This demonstrates that subthreshold resonance, mediated by I_h_ and IT, can strongly shape which input frequencies reach spike threshold — converting a band-pass impedance property into a frequency-selective spiking property.

Across resonant neurons, resonance frequency fR inversely correlates with effective input resistance Rin. Resonance can be adjusted by manipulations such as virtual constant conductance or depolarization while preserving the fR–Rin relationship, consistent with the RLC framework in which fR depends on L and C rather than R alone (Vera et al., 2020).

### 3.3 Amplitude resonance and phase-resonance

**FIGURE 3:**
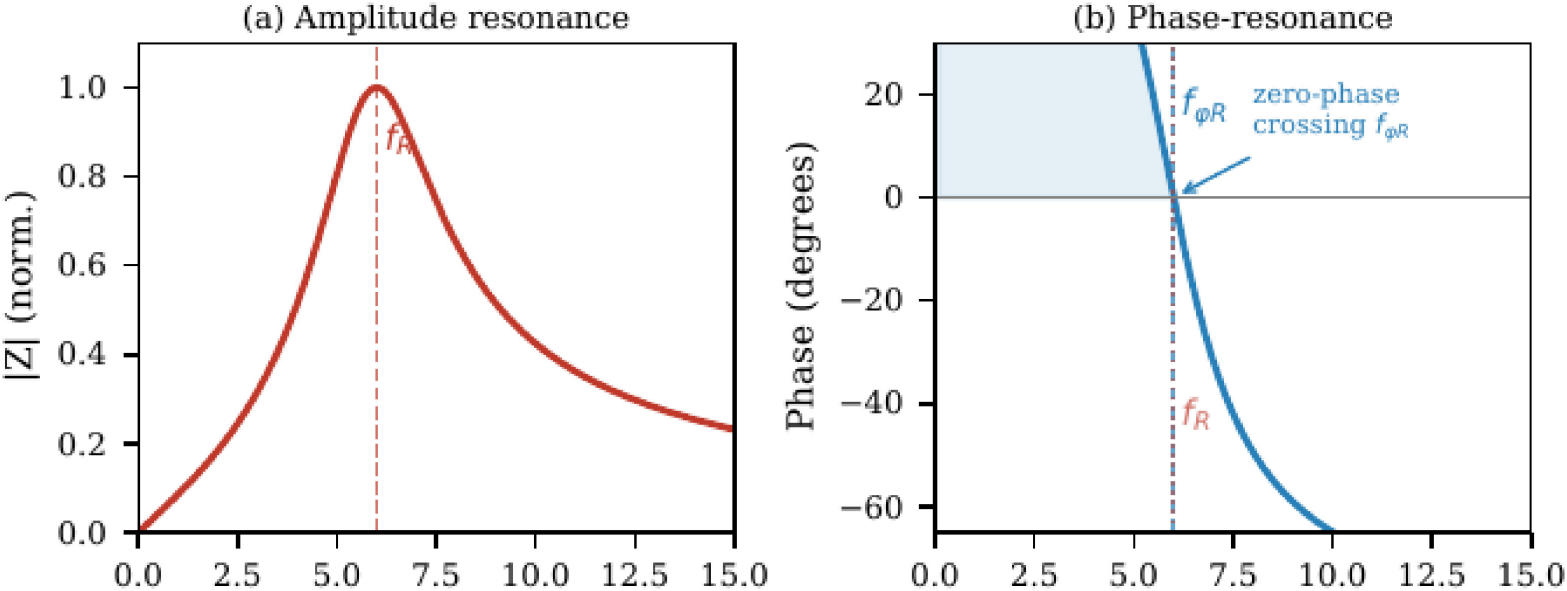
Phase-resonance versus amplitude resonance. **(a)** Impedance magnitude peaks at amplitude resonance frequency fR. **(b)** Phase-resonance occurs at fφR (the zero-phase frequency: the frequency at which the impedance phase crosses zero), which does not coincide with fR (Rotstein, 2014).

The resonance literature supports a critical distinction between amplitude resonance and phase-resonance (Rotstein, 2014) (Figure 3). Phase-resonance is the ability of neurons to exhibit a zero-phase response to oscillatory input at a non-zero frequency, distinct from the frequency of peak amplitude response. Rotstein (2014) demonstrates that these two resonance types are generated by separable biophysical mechanisms and need not coincide in frequency.

This distinction is important for the present framework because it implies that a single “resonant frequency” descriptor is insufficient. The full characterization of a resonant neuron requires at minimum: the amplitude resonance frequency fR, the phase-resonance frequency fφR, and the quality factor Q. This three-parameter description is richer than the single time constant τ of the RC model.

### 3.4 Resonance and information transfer: the non-linearity requirement

The relationship between resonance and information transmission is non-trivial, and constrains overly direct “resonance implies coding” arguments (Blankenburg et al., 2015). Blankenburg et al. (2015) demonstrated that neurons with subthreshold resonance but linear dynamics (without threshold) do not show resonance of information transfer: coherence between input and output remains low-pass, not band-pass. However, adding a threshold non-linearity — the spike — enables the impedance peak to translate into a coherence peak, producing genuine frequency-selective information transfer.

This result is foundational for the present framework. It establishes that the computational value of subthreshold resonance is not automatic: it requires non-linear transduction to convert band-pass membrane dynamics into band-pass information transfer. The spike serves this dual role — it is both a perturbation that launches resonant transients (section “4 The spike as perturbation”) and the non-linear element that converts those transients into frequency-selective output.

Richardson et al. (2003) provide complementary support. Their analysis of the transition from subthreshold resonance to firing-rate resonance shows that the frequency preference of the membrane’s impedance profile can be inherited by the neuron’s firing-rate response, but only through the mediating effect of the spike threshold. The firing-rate resonance frequency tracks the subthreshold resonance frequency, establishing a causal chain from impedance parameters through membrane resonance to output frequency preference.

The Izhikevich resonate-and-fire model (Izhikevich, 2001) provides the dynamical-systems perspective. Neurons whose subthreshold dynamics are governed by a pair of complex conjugate eigenvalues — the mathematical signature of an underdamped RLC element — exhibit qualitatively different spiking behavior from integrate-and-fire neurons: they show frequency preference, post-inhibitory rebound, and resonant amplification of periodic inputs. This model demonstrates that the distinction between RC (real eigenvalues, monotonic decay) and RLC (complex eigenvalues, oscillatory ringdown) has direct consequences for spike generation and neural coding.

Together, these results — information-theoretic (Blankenburg et al., 2015), firing-rate (Richardson et al., 2003), and dynamical-systems (Izhikevich, 2001) — converge on a single conclusion: subthreshold resonance is computationally consequential, but only through non-linear transduction. This constrains the present framework: the computational claims of section “4 The spike as perturbation” apply specifically to the regime where resonant dynamics interact with spike threshold, not to linear subthreshold dynamics in isolation.

## 4 The spike as perturbation

### 4.1 Impulse response interpretation

The spike-as-perturbation framework does not contest the canonical role of spikes as long-range communication events. Rather, it reinterprets spikes as impulse perturbations that additionally launch a post-perturbation trajectory in the postsynaptic membrane whose structure depends on the receiving neuron’s resonant parameters and its state at the moment of perturbation.

We use post-perturbation ringdown, rather than post-spike ringdown, as the primary term, because the perturbing event can take three biophysically distinct forms with decreasing safety of the impulse idealization. (i) A postsynaptic perturbation: an incoming EPSC or IPSC displaces the resonant membrane; this is the safest case, and the Dirac-impulse treatment of Equations 4, 5 is an approximation valid when the synaptic timescale is short compared with the resonant period (for fast AMPA/GABA-A kinetics versus 1–20 Hz resonance this holds). (ii) A dendritic perturbation from a backpropagating action potential: plausible but complicated by dendritic filtering and active conductances. (iii) A post-reset somatic ringdown following the neuron’s own spike: the most model-dependent, because reset, afterhyperpolarization, and refractory conductances are not captured by a small-signal linearization. The claims of this paper are stated for case (i); cases (ii) and (iii) are noted where relevant but are not the basis of the core argument.

One constraint frames everything that follows. The framework does not claim that phase exists as a usable code whenever an RLC equation can be fitted to a membrane. It claims usable phase only inside a finite readability window set by the envelope amplitude, the background fluctuation level, the quality factor, and the resonance frequency; the formal condition is stated in section “4.6 Sensitivity analysis: latency, synaptic jitter, and frequency tuning” and tested under *in vivo*-like bombardment in section “8.2 What must be hedged.” The representational comparison that follows is therefore a statement about what state variables are available in principle, not a guarantee that they are decodable under all biological conditions.

In RC models, the spike resets the membrane voltage and the response decays exponentially toward rest — one shape, one time constant, one parameter. In the RLC interpretation, the spike perturbs a resonant system, launching a transient trajectory that oscillates at ω_d and decays at rate α. This trajectory carries circuit-identity information (α, ω_d) and, when perturbing an ongoing oscillation, perturbation-timing information (φ) — a richer post-perturbation state than the RC case provides.

This framing is motivated by the effective parallel RLC description of resting impedance and its associated natural frequency (Mauro et al., 1970). The interpretation is conditional on state: at relatively hyperpolarized levels the impedance degenerates to an RC system and the perturbation produces a standard exponential decay (Mauro et al., 1970). The spike-as-perturbation framework applies specifically in the resonant regime and is not claimed to apply universally.

### 4.2 Topology and parameter mapping

The parallel RLC topology is consistent with the phenomenological decomposition in which the resting impedance consists of multiple parallel structures, yielding an effective parallel R, L, C system with a natural frequency (Mauro et al., 1970). This motivates mapping circuit parameters to functional roles: R governs energy dissipation and sets the bandwidth of the resonant peak; L (the effective inductance from voltage-gated conductance kinetics) governs reactance and sets the natural frequency; C is the membrane capacitance, the best-conserved electrical parameter across cell types (Gentet et al., 2000).

### 4.3 Representational comparison: RC vs. RLC post-perturbation trajectories

**FIGURE 4:**
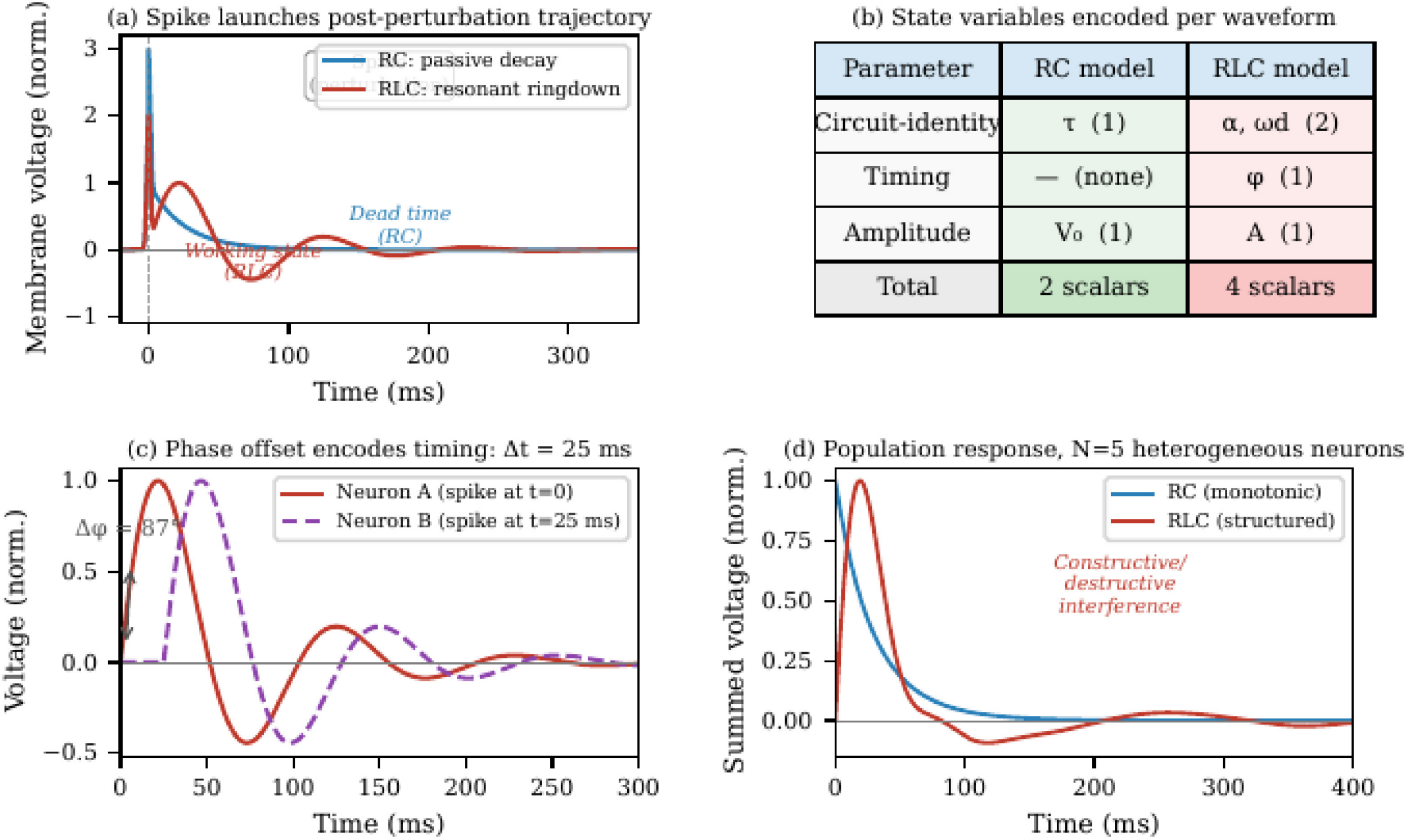
The spike-as-perturbation framework. **(a)** RC exponential decay vs RLC damped oscillation with zero-crossings. **(b)** Information content: a single matched first-order RC element carries timing only through a monotonic amplitude/slope trajectory and lacks an intrinsic phase coordinate, whereas the RLC ringdown carries circuit-identity and timing parameters. The panel labels refer to this single-element comparison; RC networks with added delays, recurrence, or adaptation can encode timing by other means. **(c)** Phase offset φ encodes perturbation timing relative to ongoing resonant state. **(d)** Population comparison.

To make the additional state-space dimension concrete (Figure 4), we distinguish two kinds of state information carried by a post-perturbation waveform: circuit-identity information (what kind of resonant element was perturbed) and perturbation-timing information (when did the perturbation arrive relative to the ongoing state). The RC response carries the first in a single parameter (τ); the RLC response carries both in a richer parameter set (α, ω_d, φ). The comparison below is a parameter-counting argument establishing that the RLC waveform is a richer representational substrate; formal mutual-information bounds would require additional assumptions about noise and the prior distribution of perturbation times.

#### 4.3.1 Single-neuron level

An RC membrane responding to an impulse produces *v*(*t*) = *V*_*0*_ e^∧^(−t/τ), where τ = RC is a fixed circuit property. The waveform shape is invariant; only amplitude V_0_ varies with input strength. An ideal observer monitoring this response can extract one circuit parameter (τ) and one perturbation parameter (V_0_), for a total of two scalar values.

An underdamped RLC membrane responding from rest produces the ringdown of (4), also with a fixed waveform shape for a given circuit. However, when the perturbation arrives during ongoing oscillatory activity — the biologically relevant case for neurons receiving continuous synaptic bombardment — the response follows (5). An ideal observer can now extract two circuit-identity parameters (α, ω_d), one amplitude parameter (A), and one perturbation-timing parameter (φ), for a total of four scalar values. The RLC post-perturbation trajectory therefore admits a richer low-dimensional parameterization than the RC case — preserving a broader set of potentially decodable transient features through the addition of a phase coordinate in addition to amplitude.

#### 4.3.2 Population level

The encoding richness is amplified across heterogeneous populations. Consider N neurons receiving a common perturbation. In an RC population with heterogeneous time constants τ_*i*_, each neuron responds with V_0_ e^∧^(−t/τ_*i*_) — N exponential decays differing only in rate. The population response is a sum of exponentials: monotonic, smooth, and rapidly convergent to a single dominant decay mode.

In an RLC population with heterogeneous (α_*i*_, ω_{d,i}), the same perturbation produces N ringdowns at different frequencies and decay rates. The population response now has temporal structure absent in the RC case: zero-crossings at neuron-specific times, constructive and destructive interference between oscillatory components, and a time-varying spectral signature that evolves as different frequency components decay at different rates. This additional temporal structure is the population-level analogue of the single-neuron representational enrichment described above.

The number of zero-crossings provides a minimal quantification. An RC neuron’s post-perturbation response has zero zero-crossings (monotonic decay). An RLC neuron’s ringdown produces approximately 2Q/π zero-crossings per e-folding time of the envelope. At biological Q ≈ 2, this yields roughly 1.3 zero-crossings per time constant — modest but non-trivial additional temporal structure per neuron.

### 4.4 A computational primitive: phase-based temporal discrimination

The preceding comparison establishes that RLC populations carry richer temporal structure than RC populations. This subsection demonstrates a concrete computational primitive — temporal discrimination via phase — that emerges naturally from RLC dynamics and is not preserved in the same intrinsic phase-coded form in matched first-order RC models. We use “subthreshold dynamical processing” here to mean the transformation of input timing into a structured trajectory variable that downstream readout can exploit.

#### 4.4.1 Setup

Consider two neurons, A and B, with identical RLC parameters (same α, ω_d), receiving the same synaptic input but with a small temporal offset Δt between them.

#### 4.4.2 RC case

Both neurons respond with exponential decays: v_A(t) = V_0_ e^∧^(−t/τ) and v_B(t) = V_0_ e^∧^(−(t−Δt)/τ). At any readout time t, the difference between the two responses is a scalar amplitude ratio: v_A(t)/v_B(t) = e^∧^(−Δt/τ). In principle, timing information is available, but it is encoded only in an exponentially decaying amplitude difference that rapidly becomes unreadable as both signals converge to zero.

#### 4.4.3 RLC case

Both neurons produce damped oscillatory ringdowns: v_A(t) = A e^∧^(−αt) sin(ω_d t) and v_B(t) = A e^∧^(−α(t−Δt)) sin(ω_d (t −Δt)). The phase difference between the two responses is Δφ = ω_d ⋅Δt, and this phase difference is preserved throughout the oscillatory portion of the ringdown. Unlike the RC case, where timing information is locked to amplitude and decays, the RLC phase difference is a geometric quantity that persists independently of amplitude until the envelope reaches noise floor.

#### 4.4.4 Frequency-dependent discrimination

The phase encoding of temporal offset scales with ω_d. At theta-range resonance (f_d ≈ 5 Hz, ω_d ≈ 31 rad/s) with Q ≈ 2, the phase discrimination resolution is approximately Δt_min ≈π/(ω_d ⋅ Q) ≈ 50 ms — the timescale of one half-oscillation period. This is relevant for hippocampal sequence encoding and theta-phase precession, where temporal offsets of 20–100 ms carry information about spatial position (Lisman and Jensen, 2013; O’Keefe and Recce, 1993). At beta-range resonance (f_d ≈ 20 Hz, ω_d ≈ 126 rad/s) with Q ≈ 2, the resolution improves to Δt_min ≈ 12 ms, entering the regime relevant for sensorimotor timing. At the high end of the biological resonance range (f_d ≈ 40 Hz, gamma-adjacent), Δt_min ≈ 6 ms — competitive with synaptic coincidence detection windows.

This example is deliberately minimal. It shows that even two identical RLC neurons with a single coincidence detector can perform a computation (phase-based temporal discrimination) that is not preserved in the same intrinsic phase-coded form in a matched first-order RC network — where timing information is encoded solely in the decaying amplitude of a monotonic exponential, rather than in a persistent geometric phase angle. The advantage is not merely quantitative (better precision) but qualitative (a different encoding dimension) — though more elaborate RC-based networks incorporating delays or recurrent structure might achieve related discriminations through different mechanisms.

### 4.5 Simulation: temporal sensitivity of RC vs. RLC ringdowns

#### 4.5.1 Methods

All simulations were implemented in Python 3.11 using NumPy and SciPy. Membrane voltages were computed analytically from the closed-form impulse-response expressions (Equations 4, 5); no numerical ODE integration was required. The RC baseline used τ_RC = 25 ms. RLC membranes were parameterized by natural frequency f_0_ = 10 Hz (panels a–c) or a range of 5–16 Hz (panel d, *N* = 5 neurons) and quality factors Q ∈{1.0, 1.5, 2.0, 3.0}. Decay rate α = π f_0_/Q and damped frequency ω_d = √((2π f_0_)^2^ −α^2^) were derived from these parameters. Temporal sensitivity was computed as |∂Δv/∂Δt| evaluated at Δt = 1 ms using a finite-difference approximation with step 10^−4^ ms. Additive Gaussian noise with σ = 0.25 (normalized units, where the normalization reference is the peak amplitude of the clean RLC ringdown = 1) was applied independently to each sample in the noisy discrimination panels. All figures were generated with Matplotlib 3.8. The simulation parameters are collected in Table 1. Code and figure-generation scripts are available from the authors and will be deposited in a public repository on acceptance.

**TABLE 1:** Simulation parameters used in section “4 The spike as perturbation.”

Parameter	Value	Used in	Biological justification
τ_RC	25 ms	RC baseline	Membrane time constant range, 10–30 ms (Koch, 1999)
f_0_	10 Hz	Main RLC comparison	Theta/alpha-range subthreshold resonance (Hutcheon et al., 1996; Ulrich, 2014)
Q	1–3	Core biological regime	Reported in resonant cortical, thalamic, hippocampal neurons (Rotstein, 2014; Vera et al., 2020)
Q	5–8	Exploratory only	High-Q visualization and sensitivity bounds, not the central claim
V_th	−55 mV	Threshold simulations (section “4.6 Sensitivity analysis: latency, synaptic jitter, and frequency tuning,” “5 Readout — subthreshold trajectories become spike-timing modulation”)	Representative spike threshold; model assumption
V_pert	8–12 mV	Input train (section “4.6 Sensitivity analysis: latency, synaptic jitter, and frequency tuning”)	Subthreshold per-input amplitude; model assumption
noise σ	0.25 (norm.)	Phase discrimination (section “4.5 Simulation: temporal sensitivity of RC vs. RLC ringdowns”)	Simulation stress test, relative to peak ringdown amplitude = 1

#### 4.5.2 Results

**FIGURE 5:**
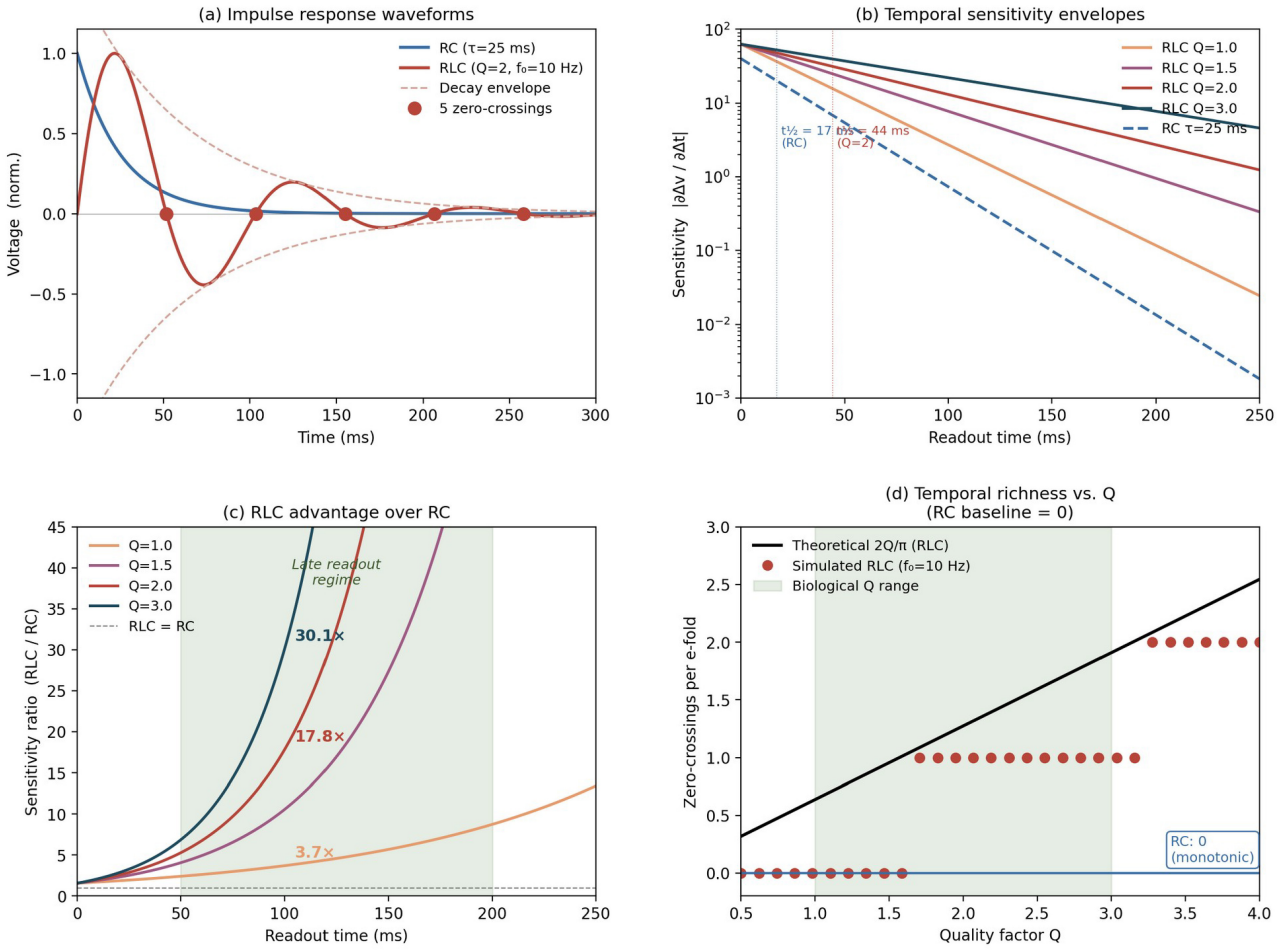
Simulation: RC vs. RLC post-perturbation dynamics. **(a)** Single-neuron impulse response: RC monotonic decay vs. RLC damped oscillation with zero-crossings. **(b)** Temporal sensitivity |∂Δv/∂Δt| vs. readout time (log scale). RLC sensitivity envelope decays more slowly than RC by a factor dependent on Q. **(c)** Sensitivity ratio (RLC/RC) vs. readout time. **(d)** Temporal richness vs. Q: theoretical 2Q/π zero-crossings per e-folding time, with simulated RLC overlay. Parameters: f_0_ = 10 Hz (panels a–c); τ_RC = 25 ms.

The preceding analytical comparison is rendered concretely in Figure 5 by evaluating the closed-form expressions (4)–(5): panels (a)–(c) are exact evaluations of the analytic sensitivity envelopes, not an independent numerical experiment, whereas the integration-based simulations of section “4.6 Sensitivity analysis: latency, synaptic jitter, and frequency tuning,” “5 Readout — subthreshold trajectories become spike-timing modulation,” “7 Population dynamics: non-linear transduction” constitute the separate numerical tests. Figure 5 presents four analyses of post-perturbation dynamics comparing RC and RLC membranes. Panel (a) shows single-neuron impulse responses: the RC membrane produces a monotonic exponential decay, while the RLC membrane (*Q* = 2, f_0_ = 10 Hz) produces a damped oscillation with multiple zero-crossings. Panel (d) shows that a heterogeneous population of *N* = 5 RLC neurons with different resonant frequencies (5–16 Hz) produces a summed response with rich temporal structure — constructive and destructive interference between oscillatory components — absent in the corresponding RC population sum, which converges monotonically to a single dominant decay mode.

Panel (b) quantifies temporal sensitivity: the magnitude of the derivative |∂Δv/∂Δt| of the difference signal between two offset responses, plotted as a function of readout time. This measures how precisely a downstream neuron could discriminate the temporal offset Δt at a given latency. The RC sensitivity envelope decays with half-life τ⋅ ln 2 ≈ 17.3 ms. The RLC sensitivity envelope decays with half-life ln 2/α = Q ⋅ ln 2/(π f_0_): 22.1 ms at *Q* = 1, 44.1 ms at *Q* = 2, and 66.2 ms at *Q* = 3. At biological Q ≈ 2, temporal information persists 2.5× longer than in the matched RC case.

**FIGURE 6:**
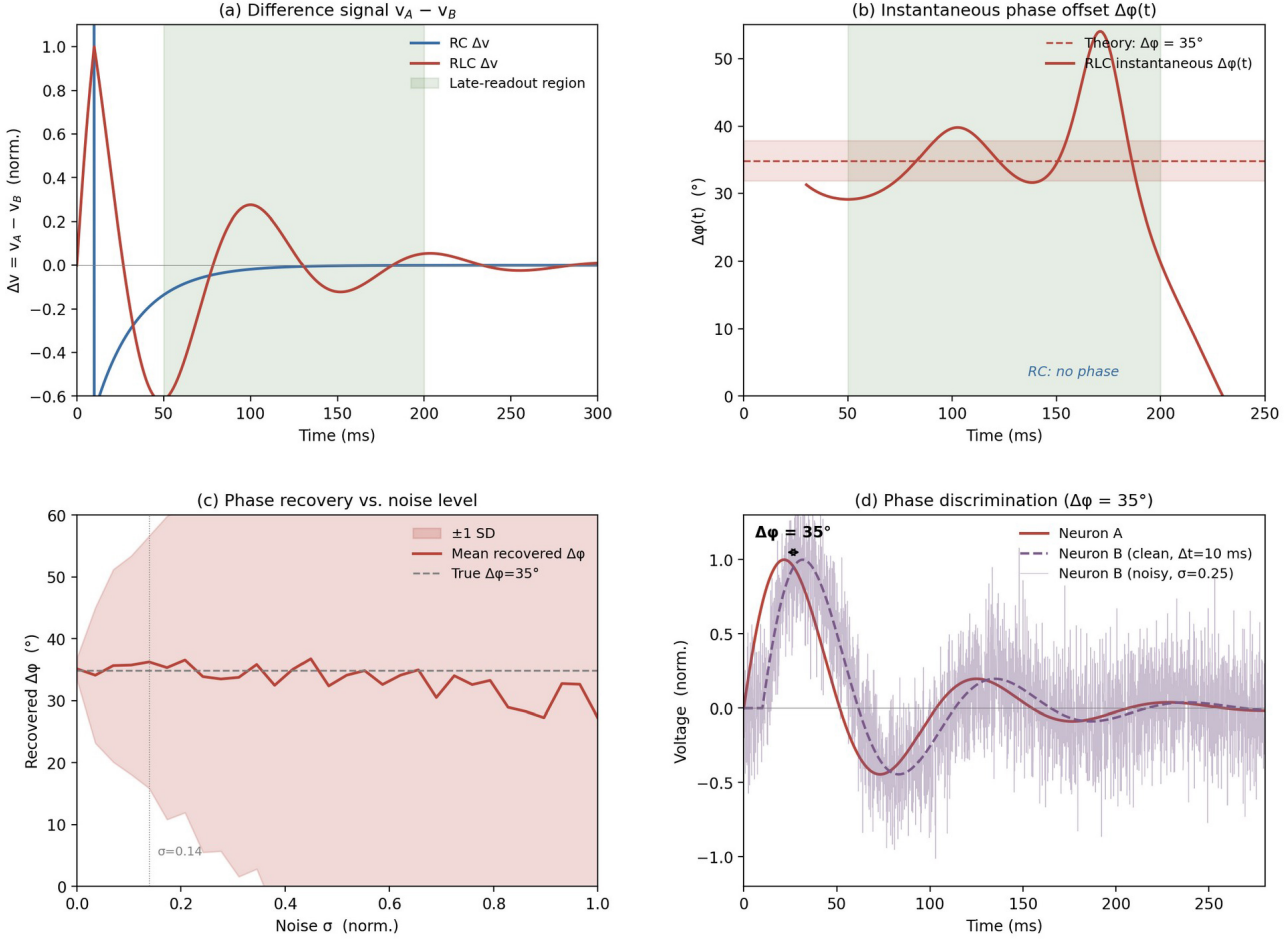
Phase discrimination analysis. **(a)** Difference signal Δv = v_A - v_B for RC and RLC, Δt = 10 ms: RLC difference is sustained and oscillatory; RC collapses within 50 ms. **(b)** Instantaneous phase offset Δφ(t) via analytic signal; RLC plateaus at 35°; RC phase undefined. **(c)** Noise robustness (peak-time readout, 500 trials per σ): mean recovered Δφ with ± 1 SD. **(d)** Discrimination traces: clean and noisy (σ = 0.25) Neuron B relative to Neuron A.

Panel (c) shows the sensitivity ratio (RLC envelope/RC) as a function of readout time. At 100 ms post-stimulus, the RLC sensitivity exceeds RC by a factor of 3.7× at *Q* = 1, 17.8× at *Q* = 2, and 30.1× at *Q* = 3. This ratio grows with readout latency because the RLC envelope decays more slowly than the RC envelope when Q > 1; it is a deterministic consequence of the two envelope half-lives evaluated at a fixed readout latency, so its magnitude is set by the choice of latency and is reported as an analytic property rather than an independent simulation outcome. The green shaded region indicates the late-readout regime (50–200 ms) where the RLC advantage is most pronounced — precisely the timescale of theta-band processing and hippocampal sequence encoding; recoverability across this window is ultimately bounded by noise (Figure 6c).

Figure 6 presents the phase discrimination analysis. Panel (a) shows the difference signal Δv(t) = v_A(t) − v_B(t) for RC and RLC neurons with Δt = 10 ms; the RLC difference signal remains oscillatory and structured throughout the late-readout regime (shaded), while the RC difference collapses within ∼50 ms. Panel (b) shows the instantaneous phase offset Δφ(t) of the RLC response computed via the analytic signal; the phase plateaus at ≈ 35° throughout the valid envelope window and is absent (undefined) for the RC case. Panel (c) quantifies noise robustness using a peak-time readout: for each noise level σ, the first-peak time difference between v_A and v_B is estimated across 500 trials. The mean recovered Δφ remains accurate below σ≈ 0.14 (normalized units); above this level the estimator variance exceeds half the signal. Panel (d) shows the raw discrimination traces: Δφ = 35° is apparent in the clean trace and identifiable in the noisy trace (σ = 0.25).

### 4.6 Sensitivity analysis: latency, synaptic jitter, and frequency tuning

**FIGURE 7:**
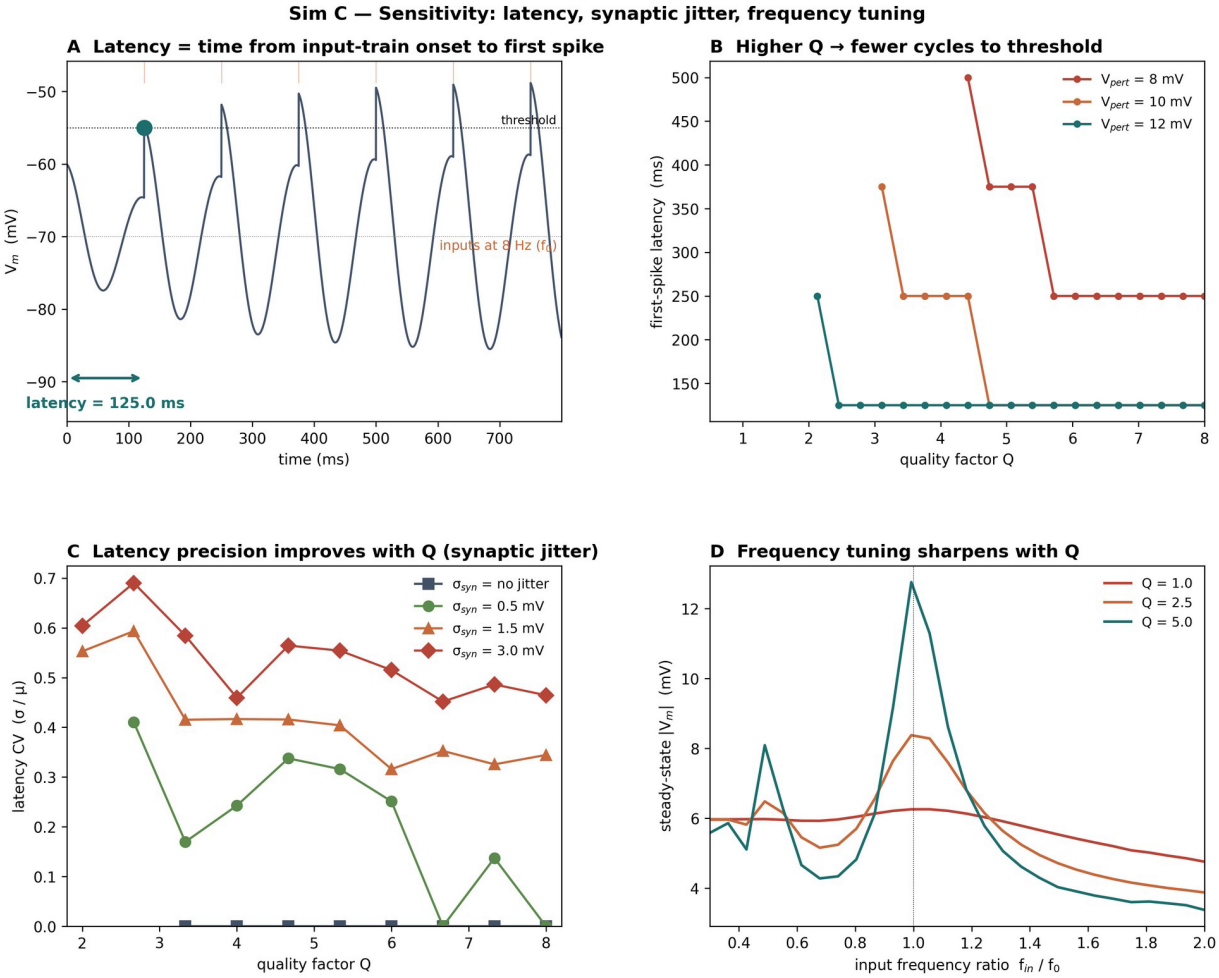
Sensitivity analysis. **(A)** Latency definition: time from input-train onset to first threshold crossing (example: *Q* = 5, V_pert = 10 mV, latency = 125 ms). **(B)** Latency vs Q at three input amplitudes; higher Q produces shorter latency, with a minimum Q below which the neuron does not fire. **(C)** Latency CV under synaptic jitter (50 trials per condition); precision improves with Q. **(D)** Frequency tuning sharpens with Q; peak amplitude rises from 6.3 mV at *Q* = 1 to 12.8 mV at *Q* = 5.

A formal sensitivity analysis quantifies robustness to noise, parameter perturbations, and synaptic fluctuations. Figure 7 provides this across four panels using a complementary integration-based protocol that drives a parallel-RLC membrane to threshold with a subthreshold input train.

We define first-spike latency as the time from input-train onset (*t* = 0) to the first crossing of threshold V_th = −55 mV (panel A). The example trace shown is a *Q* = 5 neuron driven by a subthreshold input train at f_in = f_0_ = 8 Hz with V_pert = 10 mV per input. The membrane builds up resonantly and crosses threshold on the second input cycle, giving a latency of 125 ms.

Panel B sweeps latency against Q at three input amplitudes (V_pert = 8, 10, 12 mV). Higher Q produces shorter latency at a given amplitude, consistent with the resonant build-up requiring fewer cycles to accumulate enough energy to cross threshold. Below a minimum Q the neuron does not fire at all within the 1.5-s simulation window; this minimum Q is 4.4 at V_pert = 8 mV, 3.1 at 10 mV, and 2.1 at 12 mV. The dependence on Q is monotonic across the biologically relevant range.

Panel C quantifies latency precision under synaptic fluctuations. We add trial-to-trial Gaussian jitter to V_pert with standard deviations of 0, 0.5, 1.5, and 3.0 mV (50 trials per condition) and compute the coefficient of variation of first-spike latency. CV decreases with Q at every jitter level above zero. At *Q* = 6 with synaptic jitter of 0.5 mV, CV is 0.25; at 3.0 mV, CV rises to 0.52. The neuron remains a faithful timing detector under biologically realistic input variability provided Q exceeds approximately 3.

Panel D shows steady-state membrane amplitude as a function of input frequency ratio f_in/f_0_ at three *Q*-values. The tuning curve sharpens markedly with Q. Peak amplitude at f_in = f_0_ rises from 6.3 mV at *Q* = 1 to 12.8 mV at *Q* = 5. The full-width-at-half-maximum narrows accordingly. The high-Q tuning curve shows a subharmonic peak at f_in/f_0_ ≈ 0.5; this is the expected response when every second input pulse arrives in phase with the resonance and provides an additional experimental signature of the second-order dynamics.

These results show that the modeled mechanism is robust to several biologically motivated noise and parameter perturbations within the assumed regime, that the qualitative behavior does not depend on a fine-tuned choice of Q, and that the model predicts a quantitative dependence of spike timing on Q that is directly measurable in patch-clamp recordings.

A readability condition makes this measurable rather than assumed. The phase of a ringdown is recoverable by a downstream readout only while its envelope remains above the background synaptic fluctuation floor, i.e., while A⋅e^∧^(−αt) > k⋅σ_bg for a detection factor k of order unity, which bounds the usable window to roughly t_read < (1/α)⋅ln(A/kσ_bg) = (Q/π f_0_)⋅ln(A/kσ_bg). The framework therefore predicts a readable transient only when the perturbation amplitude clears the fluctuation floor by enough to span at least a fraction of a resonant cycle; below that, the trajectory is dominated by noise and the phase variable is not defined. This condition also exposes the principal *in vivo* caveat. In the high-conductance state of active cortex, intense background bombardment lowers the effective input resistance R, and because *Q* = R√(C/L) this depresses Q toward the overdamped RC limit at the same time as it raises σ_bg. The phase-readout regime is thus expected to be expressed most strongly in quiescent or down-state epochs, in cell types with high input resistance, and under neuromodulatory conditions that elevate I_h_-dependent Q, and to be suppressed under sustained high-conductance bombardment — a state-dependence that is itself a testable signature rather than a hidden assumption.

## 5 Readout — subthreshold trajectories become spike-timing modulation

A natural objection to the framework is that downstream neurons see only spikes, not the subthreshold voltage. If the post-perturbation ringdown is invisible to the network, then any computation it performs is private. This objection requires a direct answer.

The answer is that the subthreshold trajectory does not need to be transmitted. It modulates the timing of the next spike, and the next spike IS the broadcast. The post-perturbation trajectory determines, at every moment, how close the membrane sits to threshold. An input arriving when the trajectory is near its positive peak crosses threshold sooner than the same input arriving near a trough. The subthreshold ringdown shapes a moving probability of firing. Spike timing is the network-visible readout of that hidden state.

**FIGURE 8:**
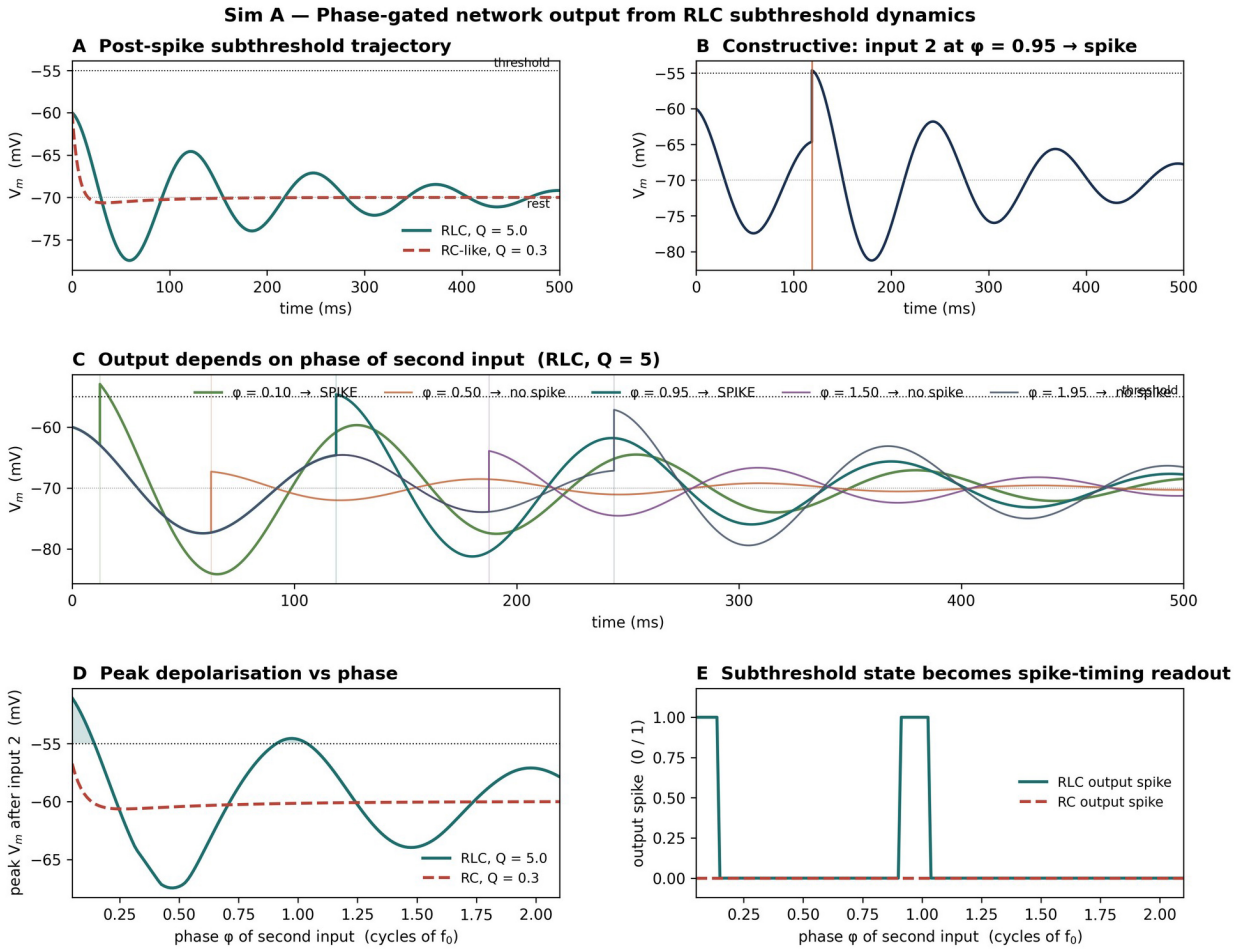
Two-spike phase-readout from RLC subthreshold dynamics. A first input drives an RLC (*Q* = 5) or RC-like (*Q* = 0.3) membrane to a fixed perturbation amplitude; a second input arrives at variable phase across the resulting subthreshold trajectory. **(A)** Post-spike subthreshold trajectory for the RLC (*Q* = 5) versus RC-like (*Q* = 0.3) membrane. **(B)** A second input arriving near the trajectory peak (phase ≈ 0.95) drives the membrane across threshold and evokes a spike. **(C)** Membrane traces for a second input delivered at several phases, showing that the spike or no-spike outcome depends on arrival phase. **(D)** Peak depolarisation after the second input versus arrival phase, showing phase-dependence for the RLC but not the RC membrane. **(E)** Binary output (spike or no-spike) versus phase: the RLC fires only in select phase windows, while the RC membrane never fires. The phase information in the subthreshold trajectory is converted into a spike or no-spike decision and broadcast through the network’s spike code.

**FIGURE 9:**
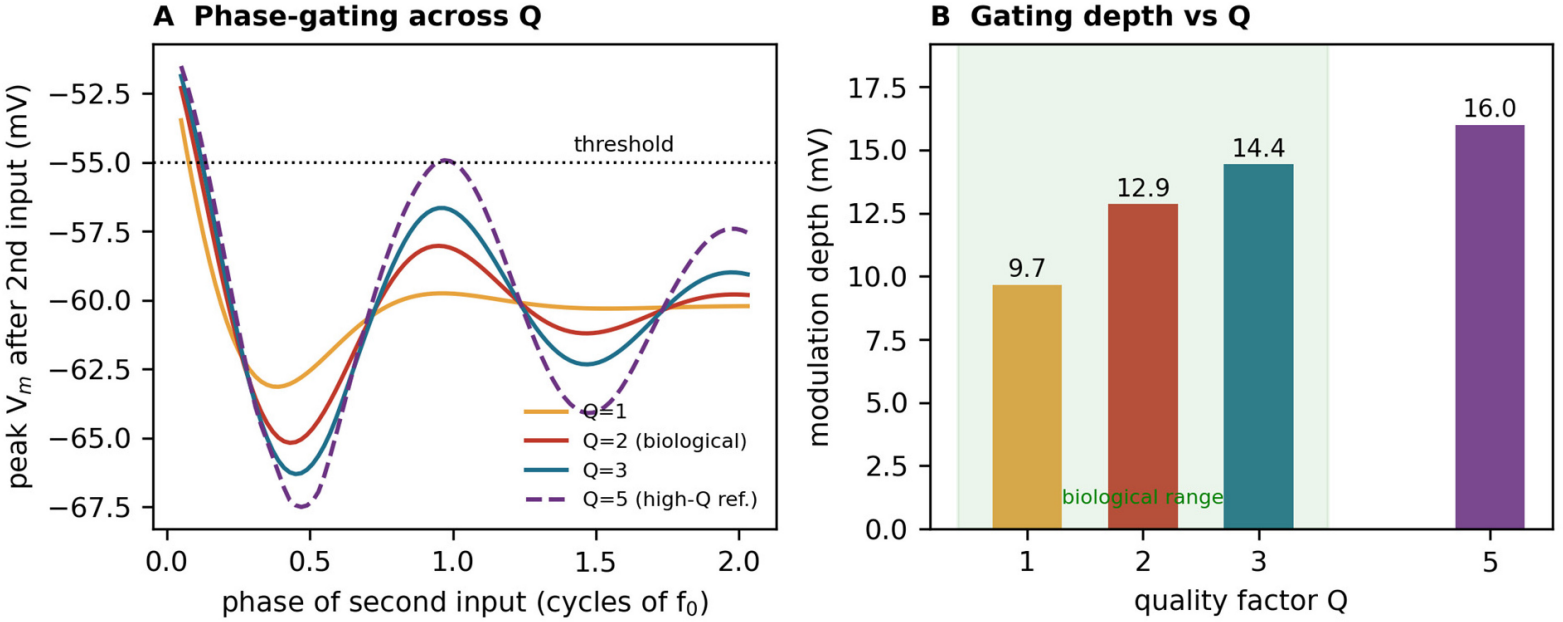
Two-spike phase-gating across the biological Q range. A first impulse launches a ringdown; a second impulse of equal amplitude arrives at variable phase. **(A)** Peak depolarization reached after the second input, versus its arrival phase, for *Q* = 1, 2, 3 and a *Q* = 5 reference; the dotted line is spike threshold. **(B)** Modulation depth — the peak-to-peak range of **(A)** — versus Q: 9.7 mV at *Q* = 1, 12.9 mV at the central biological value *Q* = 2, and 14.4 mV at *Q* = 3, rising to 16.0 mV at *Q* = 5 (RC reference *Q* = 0.3: 5.8 mV). The phase-gating is present throughout the biological range, not only at high Q; per-input amplitude calibrated so the *Q* = 5 depth matches Figure 8.

We demonstrate this with a two-spike protocol. A first input drives the membrane to a fixed perturbation amplitude. A second input arrives at variable phase across the resulting ringdown. Figure 8 shows the result for a high-Q reference cell (*Q* = 5), where the second input produces a spike only in two narrow phase windows centered on the trajectory peaks; in the RC neuron with *Q* = 0.3, no firing window exists and the response is monotone in input amplitude and independent of arrival time within the subthreshold window. The high-Q case is shown because the phase-gating is visually starkest there, but it is not confined to it: Figure 9 sweeps the same protocol across the biological range, and the gating persists throughout. The modulation depth of the post-second-input peak — the peak-to-peak range as the second input is moved across the ringdown — is 9.7 mV at *Q* = 1, 12.9 mV at the central biological value *Q* = 2, and 14.4 mV at *Q* = 3, rising to 16.0 mV at the *Q* = 5 reference and falling to 5.8 mV for the RC case (*Q* = 0.3). The phase information hidden in the subthreshold trajectory is thus converted into a spike-timing decision across the whole biological Q range, not only at high Q.

This mechanism is not exotic. It is the temporal counterpart of the dynamic gain modulation that has been characterized in resonant interneurons (Hutcheon et al., 1996) and the input-output reshaping reported in inhibition-stabilized attractor networks with Q > 0.5. The contribution of the present work is to identify this gain modulation as a broadcast mechanism for otherwise-private subthreshold computation.

Three additional channels reinforce this primary mechanism. Gap junctions provide direct subthreshold coupling in cortical interneurons and the inferior olive, transmitting the ringdown waveform without translation through spikes (Devor and Yarom, 2002; Galarreta and Hestrin, 2001). Local field potentials sum the subthreshold currents of populations and can phase-lock incoming afferents (Buzsáki, 2006; Fries, 2015). Bispectral analysis of macroscale EEG and iEEG detects quadratic phase coupling of the kind predicted by sums of resonant oscillators (Wang et al., 2026), consistent with the non-linearity surviving the averaging step from single-neuron to population.

The implication for the framework is direct. Subthreshold trajectories are not private. They reach the network through three independent channels: spike timing, gap junctions, and field signatures. The first of these — spike timing — is sufficient on its own to support the temporal-discrimination primitive demonstrated in Figure 8.

## 6 Relationship to existing neuron models

The framework presented here is not a replacement for existing neuron models. It is a unifying lens that exposes a structural relationship between them and makes explicit what each one captures and discards.

Table 2 summarizes the relationship to the four most closely related bodies of work and isolates what is specific to the present account. The contribution is not a new equation set — the subthreshold dynamics are those of a second-order linear system shared with resonate-and-fire — but a reinterpretation of the post-perturbation transient as a readable state, a readout mechanism, and the identification of Q as a neuromodulatory control variable.

**TABLE 2:** Relationship to existing work and the specific contribution of this paper.

Existing work	What it covers	What this paper adds
Resonate-and-fire (Izhikevich, 2001)	Resonance shaping spike generation; complex-eigenvalue subthreshold dynamics	Reinterpretation of the post-perturbation transient as a readable state, with an explicit spike-timing readout mechanism — an interpretation and readout layer, not a new dynamical equation
Subthreshold resonance literature (Hutcheon et al., 1996; Rotstein, 2014; Vera et al., 2020)	Impedance band-pass structure and frequency preference	Phase-coded temporal discrimination carried by the post-impulse transient, with a readability condition for real neurons
Phase response curve theory	Phase shift of an oscillator produced by a perturbation	Equivalent-circuit RLC framing with the quality factor Q as an explicit, biophysically grounded memory horizon
State-space/selective sequence models (Agrawal et al., 2025; Bal and Sengupta, 2024; Gu and Dao, 2023; Huang et al., 2024; Huber et al., 2025; Rusch and Rus, 2025)	Long-memory second-order linear recurrence; pole locations set by training or fabrication	Biophysical, neuromodulatory control of the pole radius through I_h_/Q — a runtime control variable these models lack (section “6.1 The quality factor as a neuromodulatory selectivity gate”)

The leaky integrate-and-fire neuron (LIF) is the Q → 0 limit of the present model. Setting L →∞ removes the inductive branch and leaves a parallel RC circuit. The subthreshold dynamics become a first-order exponential decay; the post-spike trajectory carries no phase or frequency content. LIF is a faithful description of neurons that operate in the heavily damped regime (*Q* < 0.5). The present framework is not opposed to LIF; it contains LIF as a degenerate special case.

The resonate-and-fire neuron (Izhikevich, 2001) is the closest existing relative. Izhikevich introduced a two-variable linear subthreshold model with reset rules at threshold. The subthreshold dynamics he derives are formally equivalent to a parallel RLC. The difference between the two frameworks is one of interpretation rather than equation. Izhikevich treats the spike as the event being computed; the subthreshold dynamics are bookkeeping. We treat the subthreshold trajectory as the computation and the spike as the broadcast. The present work is the inverse-figure version of resonate-and-fire: we look at what RF discards and ask what it computes.

The practical consequence of this reframing is that spikes become Dirac-delta perturbations of a continuously evolving linear system, rather than reset events imposed from outside. The two views are not in conflict. A reset that restores V to V_reset and I_L to I_{L,reset} is mathematically a perturbation of the state vector by (V_reset − V, I_{L,reset}− I_L). Whether one writes the spike as a reset rule or a perturbation kernel is a notational choice. The substantive claim of the present work is that the post-perturbation trajectory, however parameterized, carries computational content that is recoverable by the network through the readout mechanism of section “5 Readout — subthreshold trajectories become spike-timing modulation.”

The FitzHugh–Nagumo and Hindmarsh–Rose models share the two-dimensional subthreshold structure of the RLC framework but parametrize it in terms of dimensionless fast and slow variables rather than physical voltage and inductor current. The translation is straightforward; we do not duplicate it here.

Full conductance-based models (Hodgkin–Huxley and its multi-compartment descendants) reduce to the present framework upon linearization around the resting attractor. Mauro (Mauro et al., 1970) derived the parallel RLC analogue of the squid-axon Hodgkin–Huxley equations by exactly this procedure. Hutcheon and Yarom (2000) reviewed the corresponding reduction for thalamic and cortical neurons. The present work does not displace conductance-based models. It provides the linearized small-signal description needed to expose the phase-coded computational primitive that the full non-linear models obscure behind their parameter count.

### 6.1 The quality factor as a neuromodulatory selectivity gate

The recent sequence-modeling literature has independently rediscovered the second-order linear recurrence that underlies this framework. Linear oscillatory state-space models build their hidden layer from forced harmonic oscillators and outperform leading first-order architectures on long sequences (Rusch and Rus, 2025). The resonate-and-fire neuron has been derived directly as a structured state-space model and scaled into deep spiking networks (Bal and Sengupta, 2024; Huber et al., 2025), and second-order spiking state-space layers are now an active hardware target (Agrawal et al., 2025; Huang et al., 2024). These results restate, on the engineering side, the equivalence used here: a neuron with complex-conjugate subthreshold poles is a continuous-time state-space cell. We do not claim that equivalence as novel.

What these architectures lack is a runtime control variable: their pole locations are fixed by training or fabrication. Yet the property that distinguishes selective state-space models is the input- and context-dependent adjustment of the per-step decay rate (Gu and Dao, 2023), and recent compute-in-memory work states the gap directly — a static state matrix cannot adapt to input content, and the proposed remedy is an *in situ* control signal, such as a bias voltage, that modulates the state decay rate on the fly (Zhang et al., 2026). The role of that decay rate is equally explicit: discrete eigenvalues near the unit circle yield long memory, eigenvalues near the origin yield memory selectivity (Rickenbach et al., 2025). Eigenvalue magnitude is the memory horizon.

The present framework supplies that control variable, and it is biophysical rather than learned. The continuous-time poles of the parallel RLC membrane are Equation 6:


$$s=-\alpha \pm j\,\omega \,\_\,d,\alpha =1/\left(2\,R\,C\right)=\omega_{0}/\left(2\,Q\right),\omega_{0}=1/\sqrt{\left(L\,C\right)},$$ (6)

$$\;\;Q=R\,\sqrt{\left(C/L\right)}$$

Under the discretization step Δt that any state-space implementation imposes, the discrete eigenvalue is λ = e^∧^(sΔt), with magnitude (Equation 7):


$$\left|\lambda \right|=e\,\wedge \,\left(-\alpha \,\Delta \,t\right)=e\,x\,p\,\left(-\omega_{0}\,\Delta \,t/2\,Q\right)=e\,x\,p\,\left(-\pi \,f_{0}\,\Delta \,t/Q\right)$$ (7)

The quality factor is therefore the pole-radius control. As Q rises, αΔt → 0 and |λ| → 1: the membrane approaches the marginally stable, long-memory regime. As Q → 0 the pole collapses toward the origin and the membrane reverts to the short-memory leaky integrator, recovering LIF as the no-memory limit. The 1/e transient memory horizon (a state-persistence time, not memory storage) of the post-perturbation state is Equation 8:


$$\tau \,\_\,m\,e\,m=1/\alpha =Q/\left(\pi \,f_{0}\right)$$ (8)

so the held-state lifetime scales linearly with Q — its half-life ln 2⋅τ_mem reproduces the 44 ms reported at *Q* = 2 in section “4.5 Simulation: temporal sensitivity of RC vs. RLC ringdowns” — and the number of readable ring cycles before 1/e decay is approximately Q/π, identifying Q with representational depth per element.

The control claim follows. The membrane capacitance C is the best-conserved electrical parameter of the cell (Gentet et al., 2000) and is effectively fixed, so Q is set by R and L, both under second-messenger control. The cAMP–HCN pathway tunes input resistance and the effective inductive kinetics of I_h_, allowing neuromodulators to set the membrane time constant and energy budget directly (Byczkowicz et al., 2019; Ceballos et al., 2021). Moving I_h_ therefore moves α — equivalently |λ| — on the timescale of a neuromodulatory transient. Where a selective machine model makes its effective decay rate input-dependent through a learned gate (Gu and Dao, 2023), the neuron makes its pole radius context-dependent through neuromodulatory action on Q. Q-modulation may thus provide one biophysical mechanism for state-dependent selectivity.

Two consequences distinguish this reading from a relabeling of resonate-and-fire. First, it predicts a single measurable control axis — Q, set by I_h_ — along which a population should slide between integrator-like and long-memory regimes, tied to a specific blockable channel (section “8.3 Testable predictions,” Prediction 4). Second, it carries an energetic interpretation absent from any digital state-space model: by definition Q is the ratio of stored to per-cycle dissipated energy, so the held subthreshold state is stored oscillatory tank energy, and τ_mem and energy-per-held-state are governed by the same parameter. Adiabatic readout in the high-Q regime dissipates a fraction scaling as 1/Q per access, making memory horizon and energy cost co-tunable through one knob — a tradeoff a purely numerical hidden state cannot express.

In summary, the framework sits at a specific level of abstraction. It is more detailed than LIF because it retains the second-order resonant structure. It is less detailed than conductance-based models because it linearizes around rest. It shares dynamical equations with resonate-and-fire but interprets them differently. The contribution is not a new equation set; it is the identification of post-perturbation subthreshold trajectories as a computational substrate, the readout mechanism through which that computation reaches the network, and the identification of the quality factor Q as a neuromodulatory control variable that sets the membrane’s effective memory horizon (section “6.1 The quality factor as a neuromodulatory selectivity gate”).

## 7 Population dynamics: non-linear transduction

Section “3.4 Resonance and information transfer: the non-linearity requirement” established that resonance alone does not guarantee resonant information transfer; a non-linearity is required (Blankenburg et al., 2015). In Freeman-style K-set modeling, the transformation from postsynaptic potentials to population firing rate is captured by an asymmetric sigmoid (Eeckman and Freeman, 1991; Kay, 2016; Kay and Kozma, 2017), with empirical support from chronic recordings showing a sigmoid increase in axonal firing frequency with increasing presynaptic pulse amplitude. This is the population-level non-linearity that the present framework requires.

At the population level, mutual excitation and inhibition with asymmetric sigmoid non-linearity and dispersive delays are sufficient to produce chaotic solutions and selective destabilization by input (Freeman, 1994; Kay and Kozma, 2017), establishing that the population stage supplies the non-linear transduction the framework requires. (Whether learned configurations of resonant parameters shape a trajectory landscape is a broader claim reserved for section “ 8.5 Speculative extensions and testable predictions”.).

A network-level bridge comes from Bel and Rotstein (2019), who establish that membrane potential resonance is required for emergent oscillations in certain circuits, with network frequency tracking cellular resonant frequency monotonically. Because this result is falsifiable — experimentally shifting cellular resonance should shift network oscillation frequency — it provides a concrete link between the single-cell RLC parameters described in section “2 Biophysical foundations” and the population-level dynamics that cognitive function requires.

**FIGURE 10:**
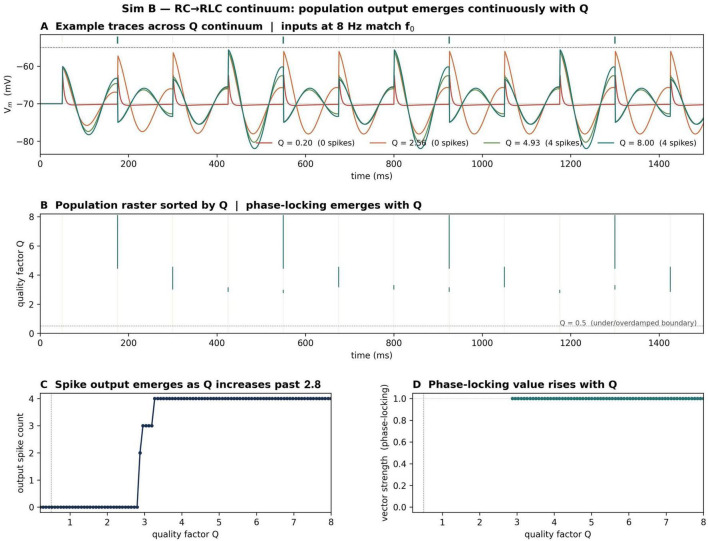
Population response across the Q continuum. 100 model neurons with Q ∈ [0.2, 8.0] receive a common 8 Hz input train (matched to f_0_). **(A)** Example membrane-voltage traces for representative Q values, with higher Q producing larger, more sustained responses. **(B)** Population spike raster sorted by Q, showing phase-locked firing emerging as Q increases. **(C)** Spike count versus Q, showing output emerging as Q rises. **(D)** Phase-locking strength (vector strength) versus Q, reaching 1.0 in the high-Q regime. Below Q ≈ 2.8 the population is silent. The transition between RC-like integration and RLC-like phase-locked computation is continuous, supporting heterogeneous networks containing neurons across the Q range.

The transition from RC-like to RLC-like behavior is continuous rather than categorical. Figure 10 shows the response of 100 model neurons spanning Q ∈ [0.2, 8.0] to a common 8 Hz input train. At low Q the population is silent; phase-locked firing emerges as Q crosses approximately 2.8, and the proportion of firing neurons rises monotonically thereafter. Vector strength reaches 1.0 in the high-Q population, indicating tight phase-locking to the input. The population thus implements a continuum of computational regimes rather than a sharp boundary, and a heterogeneous network containing neurons across the Q range can express both RC-like integration and RLC-like phase-coded computation simultaneously.

## 8 Discussion

### 8.1 What is well supported

The biophysical case rests on converging evidence from three eras of measurement. Modern channel-specific impedance analyses in mammalian CA1 pyramidal neurons show that h channels contribute a location-dependent and adaptive inductive component with theta-range negative delay, modifiable by experience and neuromodulatory state (Narayanan and Johnston, 2008). Causal resonance physiology demonstrates that manipulating I_h_ reshapes resonance profiles and links resonance to frequency-selective spiking in both cortical and thalamic preparations (Hutcheon et al., 1996; Ulrich, 2014). These contemporary results are grounded by — and consistent with — the classic axonal impedance record: a low-frequency impedance maximum, reactance vanishing between 150 and 300 Hz, membrane-localized inductance-like components, and the explicit inductive element reported by Cole and Baker (Cole and Baker, 1941; Cole and Curtis, 1939; Sabah, 2000). Taken together, these are direct measurements, not model inferences.

Second, the non-linearity requirement — resonance alone does not produce resonant information transfer without spike threshold (Blankenburg et al., 2015) — is established and is incorporated as a central constraint of the present framework.

At the network level, oscillations depend on membrane potential resonance, and network frequency monotonically depends on the resonator’s resonant frequency — a falsifiable frequency-tracking relationship (Bel and Rotstein, 2019).

### 8.2 What must be hedged

First, inductance-like behavior may be phenomenological and regime-dependent. Subthreshold behavior can arise from voltage-dependent, time-variant conductances, and impedance can degenerate to RC at hyperpolarized levels (Mauro et al., 1970).

Second, inductive reactance may reflect active conductances whose properties vary with location and activity (Cole and Baker, 1941; Narayanan and Johnston, 2008).

Third, subthreshold resonance must not be conflated with resonant information transfer. Neurons with resonant but linear subthreshold dynamics show low-pass coherence, not band-pass. Adding non-linearities enables impedance peaks to translate into coherence peaks (Blankenburg et al., 2015). The computational claims of this paper are conditioned on this non-linear regime.

Fourth, phase-resonance is distinct from amplitude resonance, and the two frequencies do not generally coincide (Rotstein, 2014). This constrains models relying on a single “resonant frequency” descriptor.

Fifth, the biological Q range (Q ≈ 1–3) places a genuine constraint on the expressiveness of the transient state language. The simulation analysis (section “ 4.5 Simulation: temporal sensitivity of RC vs. RLC ringdowns”) quantifies this constraint under the analytic envelope metric: at *Q* = 2, the sensitivity half-life is 44 ms vs. 17 ms for RC, and the model-derived sensitivity ratio at 100 ms is 17.8×. The advantage is present but operates on theta-to-beta timescales rather than millisecond coincidence detection timescales. The framework is most naturally applicable to processing at timescales commensurate with the resonant period.

Sixth, the cognitive interpretation is a Tier 3 framework proposal, not a demonstrated mechanism; see section “8.5 Speculative extensions and testable predictions.”

Seventh, and most consequentially, the clean ringdown assumed here may be an *in vitro* or quiescent-state phenomenon. In the high-conductance state of active cortex, background bombardment both raises the synaptic fluctuation floor and lowers the effective input resistance, depressing Q toward the overdamped limit (section “4.6 Sensitivity analysis: latency, synaptic jitter, and frequency tuning”). The framework does not claim that phase-coded readout operates continuously *in vivo*; it claims that the substrate is available in specific biophysical states, and it makes the boundary of that availability — the readability condition of section “4.6 Sensitivity analysis: latency, synaptic jitter, and frequency tuning” — explicit and falsifiable.

**FIGURE 11:**
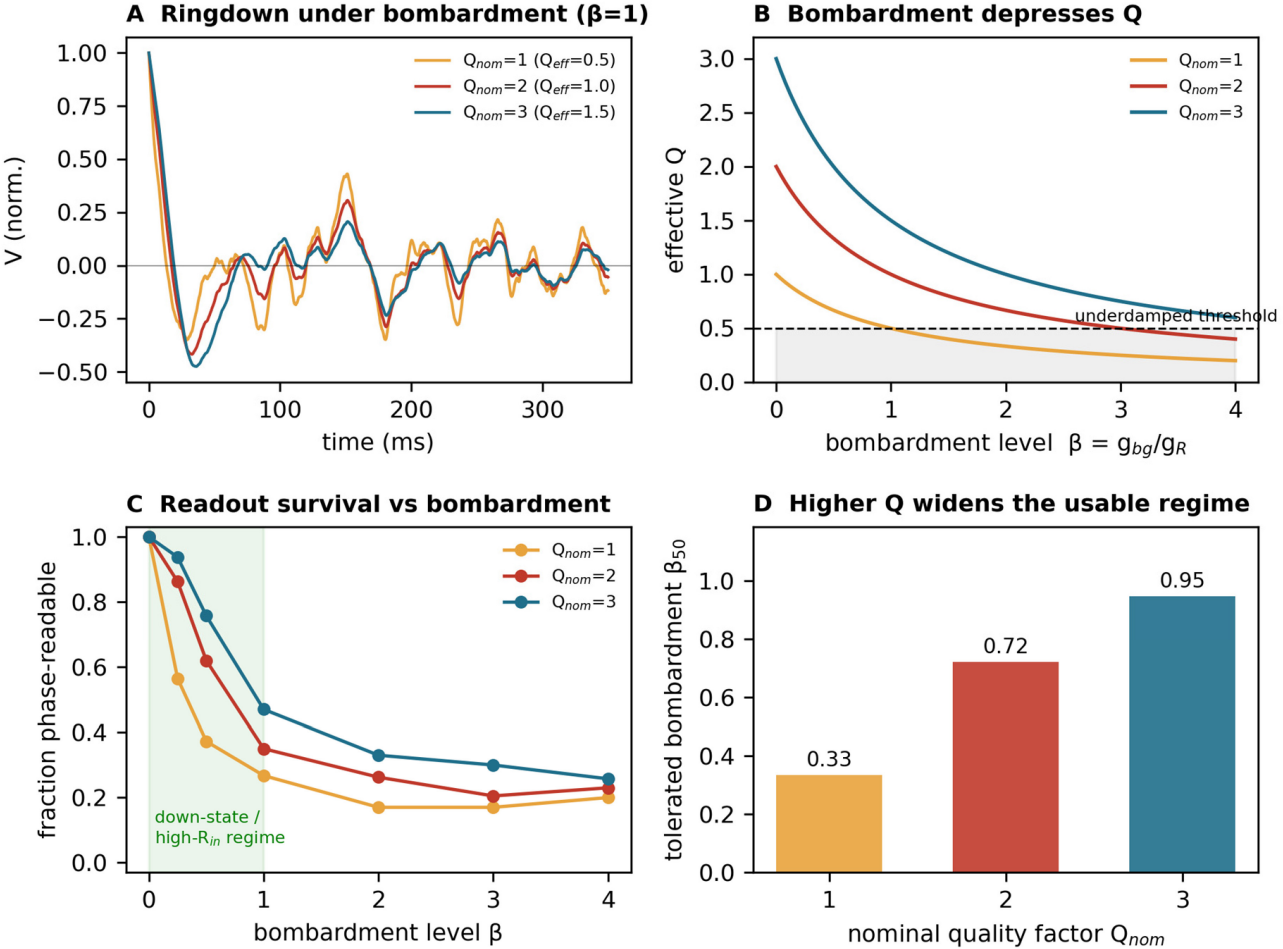
Phase-readout survival under synaptic bombardment. Background bombardment of level β = g_bg/g_R both loads the membrane (Q_eff = Q_nom/(1 + β)) and adds Ornstein–Uhlenbeck current noise. **(A)** Example ringdowns at β = 1 for Q_nom = 1, 2, 3. **(B)** Effective Q collapses with bombardment; below the dashed underdamped threshold (Q_eff = 0.5) no oscillation remains. **(C)** Fraction of trials in which a 10 ms offset is recovered to within ∼30°, versus β (400 trials per point); the shaded band marks the quiescent/high input-resistance regime. **(D)** Bombardment tolerated at half-maximal readability (β_50_); higher nominal Q widens the usable regime roughly threefold. Phase readout is reliable in low-conductance states and is suppressed under full *in vivo* bombardment.

We tested this boundary directly. Figure 11 simulates the phase-discrimination readout of section “4 The spike as perturbation” under an *in vivo*-like high-conductance state, modeled as a background bombardment of level β = g_bg/g_R that both loads the membrane — reducing the effective quality factor as Q_eff = Q_nom/(1 + β) — and injects Ornstein–Uhlenbeck synaptic current noise growing with β. Two cells receive the same impulse with a 10 ms offset; a trial is counted readable when the peak-time readout recovers that offset to within ∼30°. In the quiescent regime (β ≲ 0.5–1), corresponding to down-states and high input-resistance cells, the offset is recovered reliably and the fraction readable rises with Q. As bombardment approaches the full high-conductance state (β→ 4, total conductance several times the resonant leak), loading drives Q_eff below the underdamped threshold of 0.5 and the readout collapses toward chance for all Q. The nominal quality factor sets the width of the usable window: the bombardment level at which readability falls to one half is β_50_ ≈ 0.33, 0.72, and 0.95 for Q_nom = 1, 2, and 3 — higher Q tolerating roughly threefold more bombardment before the phase variable becomes unreadable. This confirms the central claim quantitatively while bounding it: post-perturbation phase is a usable code in resonant, low-conductance states and is suppressed under sustained high-conductance bombardment, rather than a universal *in vivo* coding scheme.

### 8.3 Testable predictions

The following predictions are ordered from most directly testable (cellular level) to most theoretically motivated. Predictions 1–3 test the biophysical substrate; Prediction 4 tests the control-law entailment that Q sets the population eigenvalue spectrum (section “6.1 The quality factor as a neuromodulatory selectivity gate”).

#### 8.3.1 Prediction 1: I_h_-dependent phase sensitivity

Selective reduction of h-channel contributions should reduce theta-range phase-leading behavior and phase-sensitive responsiveness in neurons where intrinsic phase response is attributable to h-channel properties (Narayanan and Johnston, 2008). Experimentally: ZD7288 application (a selective I_h_ blocker) should flatten the phase-frequency curve toward the monotonic phase lag characteristic of an RC membrane.

#### 8.3.2 Prediction 2: network frequency tracking

In circuits where network oscillations depend on membrane potential resonance (Bel and Rotstein, 2019), experimentally induced shifts in single-neuron resonant frequency should produce monotonic shifts in network oscillation frequency. This is testable via dynamic clamp manipulation of I_h_ in identified resonant neurons combined with LFP recording.

#### 8.3.3 Prediction 3: decoding with phase-resonance features

Decoding schemes incorporating phase-resonance — the frequency at which a neuron exhibits a zero-phase response — should separate conditions that appear similar under amplitude resonance alone, since phase-resonance provides an independent coding dimension (Rotstein, 2014). This is testable using existing multielectrode recordings with impedance characterization.

#### 8.3.4 Prediction 4 (framework-derived): Q sets the population eigenvalue spectrum and its dimensionality

If the quality factor is the membrane pole-radius control (section “6.1 The quality factor as a neuromodulatory selectivity gate”), then experimentally moving a population along the Q axis should move the eigenvalue spectrum of its collective dynamics. Concretely: elevate Q by raising cAMP or by selectively reducing fast HCN1 kinetics, and collapse it with the I_h_ blocker ZD7288 (Heys et al., 2010), while recording population activity and fitting a (switching) linear state-space model to the latent dynamics. The framework predicts that as Q rises (i) the fitted discrete eigenvalues migrate toward the unit circle, (ii) the intrinsic timescale of population autocorrelation lengthens in proportion to Q/(π f_0_), and (iii) effective dimensionality — the participation ratio, or the number of principal components needed to capture a fixed variance fraction — increases monotonically. A static rate-coding account without intrinsic resonance predicts no specific Q-linked eigenvalue migration; a static-connectivity account predicts dimensionality fixed by wiring rather than by a fast pharmacological knob. Rate models with adaptation or recurrent gain modulation can themselves produce eigenvalue shifts, so the discriminating signature is the specific scaling of the shift with Q — and its co-variation with the I_h_ manipulation — not the mere presence of a shift. The prediction is testable with existing patch-clamp and population-recording methods combined with standard state-space fitting, and it is the sharpest single point of separation between the present framework and its competitors.

### 8.4 Implications for neuromorphic engineering

If neural computation depends on resonant dynamics and adaptive coupling, neuromorphic implementations may benefit from explicit resonant elements rather than solely leaky integrators (Bel and Rotstein, 2019; Ziegler et al., 2018). A minimal resonant neuromorphic tile would consist of: (i) an LC resonator with tunable effective inductance [implementable as a gyrator circuit or voltage-controlled transconductor pair (Ziegler et al., 2018)]; (ii) a memristive synapse (Boybat et al., 2018; Indiveri et al., 2013; Rao, 2021; Wang et al., 2017; Weilenmann et al., 2024) providing the history-dependent, adaptive coupling that learning-dependent trajectory shaping requires; and (iii) a threshold comparator whose output perturbation launches the post-synaptic ringdown. The key design parameter is Q: biological Q ≈ 1–3 is achievable in analog CMOS with straightforward transconductor scaling, and the slower sensitivity decay at higher Q may translate into reduced energy expenditure per discriminated event, if implemented in hardware where Q-controlled persistence reduces the need for recurrent refresh or repeated sampling — relevant for edge-AI and implantable biosensor applications, though establishing the saving would require an explicit energy model. As a precedent for clinical scalability, Bansal et al. (2025) showed that participant-specific R, L, C values derived from MRI can reproduce whole-brain dynamics with controllability disrupted in disease, motivating RLC-based architectures as both computational and clinical tools. A single analog bias controlling Q would realize the *in situ* decay-rate control that selective state-space hardware has identified as the missing ingredient for input-dependent memory (Zhang et al., 2026) (section “6.1 The quality factor as a neuromodulatory selectivity gate”) — input-dependent selectivity from one control line rather than per-step matrix recomputation.

### 8.5 Speculative extensions and testable predictions

This subsection collects forward-looking hypotheses motivated by the framework; its empirical content is exhausted by section “2 Biophysical foundations,” “3 Evidence for inductance and resonance,” “4 The spike as perturbation,” “5 Readout — subthreshold trajectories become spike-timing modulation,” “6 Relationship to existing neuron models,” “7 Population dynamics: non-linear transduction,” and the material here represents extensions, not foundations. If perturbation-induced ringdowns implement a phase-coded transient state, these dynamics may have implications for working memory (stimulus information persisting in subthreshold phase over the Q-scaled timescale of I_h_ and IM) (Wang, 2002; Wong and Wang, 2006), temporal binding (phase coherence among resonant elements) (Singer, 1999; Varela et al., 2001), and attention (elevated Q in task-relevant populations, testable as a cholinergic, I_h_-dependent reduction in population dimensionality, distinct from rate-coding or connectivity accounts). All three remain speculative and require intracellular or population-level validation.

## 9 Conclusion

This paper has presented a spike-as-perturbation framework grounded in resonant neural biophysics, organized in three tiers of decreasing evidential support.

Tier 1: the biophysical substrate (well supported). Neural membranes exhibit frequency-dependent impedance with inductive or inductance-like components in some regimes, representable as equivalent circuits with R, L, and C elements (Cole and Baker, 1941; Fenollosa and Bisquert, 2025; Mauro et al., 1970). These resonant dynamics are generated by identifiable ion channels (Narayanan and Johnston, 2008), produce measurable subthreshold resonance (Hutcheon et al., 1996; Rotstein, 2014; Ulrich, 2014), and — critically — require non-linear thresholding to convert subthreshold resonance into frequency-selective information transfer (Blankenburg et al., 2015). The biological grounding of the equivalent-circuit reduction is now made explicit (section “2.4 Biological grounding and domain of validity”), and the domain of validity is bounded by the requirement that the membrane sit in a stable equilibrium with at least one slow current carrying a non-trivial inductive component.

Tier 2: the spike-as-perturbation interpretation (plausible inference). Beyond their canonical role as long-range signals, spikes function as impulse perturbations that launch regime-dependent resonant trajectories. These trajectories carry circuit-identity information (α, ω_d) and perturbation-timing information (φ) not available to a single matched first-order RC element without added delays, recurrence, or extra state variables. A concrete computational primitive — phase-based temporal discrimination with frequency-dependent resolution from ∼50 ms at theta to ∼6 ms at gamma-adjacent frequencies — demonstrates a qualitative capability not preserved in the same intrinsic phase-coded form in matched first-order RC models. The readout mechanism (section “5 Readout — subthreshold trajectories become spike-timing modulation”) closes the visibility gap by showing that subthreshold trajectories modulate spike timing, and the spike itself is the broadcast. A four-panel sensitivity analysis (section “4.6 Sensitivity analysis: latency, synaptic jitter, and frequency tuning”) quantifies robustness to synaptic jitter and parameter variation. The framework relates to existing neuron models as follows: LIF is the Q → 0 limit, resonate-and-fire shares the equations and differs in interpretation, and conductance-based models reduce to the present framework upon linearization around rest (section “6 Relationship to existing neuron models”).

Tier 3: the cognitive interpretation (speculative extension, section “8.5 Speculative extensions and testable predictions”). Cognition, in this framework, may be the structured evolution of perturbation-induced transient states across coupled resonant populations. The empirical content of the framework is exhausted by Tier 1 and Tier 2; the Tier 3 material is offered as testable extensions, not foundations.

The four predictions — I_h_-dependent phase sensitivity, network frequency tracking, decoding with phase-resonance features, and Q-dependent eigenvalue spectrum and dimensionality — provide routes to falsification spanning cellular, network, decoding, and population levels.

In summary, the RC model treats inter-spike membrane dynamics as a passive, informationally inert interval. The resonant perturbation framework proposes that, in regimes where inductive membrane components are active, these dynamics may instead carry decodable state information through phase, frequency, and damping structure. Whether and to what extent this potential is exploited by neural circuits remains an open empirical question — one for which this paper offers a biophysical substrate, a quantified computational primitive (under specific model assumptions), a readout mechanism making the substrate network-visible, and four falsifiable predictions — each testable with existing experimental tools.

## Data Availability

The raw data supporting the conclusions of this article will be made available by the authors, without undue reservation.
